# Bayesian uncertainty quantification for transmissibility of influenza, norovirus and Ebola using information geometry

**DOI:** 10.1098/rsif.2016.0279

**Published:** 2016-08

**Authors:** Thomas House, Ashley Ford, Shiwei Lan, Samuel Bilson, Elizabeth Buckingham-Jeffery, Mark Girolami

**Affiliations:** 1 School of Mathematics, University of Manchester, Oxford Road, Manchester M13 9PL, UK; 2 Warwick Infectious Disease Epidemiology Research Centre (WIDER), Warwick Mathematics Institute, University of Warwick, Gibbet Hill Road, Coventry CV4 7AL, UK; 3 Department of Statistics, University of Warwick, Gibbet Hill Road, Coventry CV4 7AL, UK; 4 Complexity Science Doctoral Training Centre, University of Warwick, Gibbet Hill Road, Coventry CV4 7AL, UK; 5 School of Mathematics, University of Bristol, Bristol BS8 1TW, UK

**Keywords:** shedding, Markov chain Monte Carlo, compartmental model

## Abstract

Infectious diseases exert a large and in many contexts growing burden on human health, but violate most of the assumptions of classical epidemiological statistics and hence require a mathematically sophisticated approach. Viral shedding data are collected during human studies—either where volunteers are infected with a disease or where existing cases are recruited—in which the levels of live virus produced over time are measured. These have traditionally been difficult to analyse due to strong, complex correlations between parameters. Here, we show how a Bayesian approach to the inverse problem together with modern Markov chain Monte Carlo algorithms based on information geometry can overcome these difficulties and yield insights into the disease dynamics of two of the most prevalent human pathogens—influenza and norovirus—as well as Ebola virus disease.

## 1. Introduction

Infectious diseases continue to pose a major threat to human health, with transmission dynamic models playing a key role in developing scientific understanding of their spread and informing public health policy [1]. These models typically require many parameters to make accurate predictions, which interact with data in complex, nonlinear ways. It is seldom possible to perform a series of individual controlled experiments to calibrate these models, meaning that the use of multiple datasets is often necessary [2].

At the population level, infectious disease models most frequently involve a ‘compartmental’ approach in which individuals progress between different discrete disease states, usually at constant rates [3]. We note that alternatives to such a compartmental approach exist, for example, use of a deterministic time-varying infectivity, or allowing for non-Markovian state transitions in a stochastic context [4]. There are theoretical differences between these and compartmental models for predictive epidemic modelling (see [5] for additional discussion), but from the point of view of inference they pose very similar challenges and therefore the rest of this paper will consider a compartmental modelling framework.

At the individual level, there are three separate events that can be represented using different compartments: (i) an individual receiving an infectious dose of a pathogen, (ii) the individual becoming infectious and able to infect new cases, and (iii) the individual ceasing to be infectious and becoming unable to infect new cases. For some pathogens, there are also events relating to changes in disease progression during the infectious period. The times elapsed between these events are important for understanding the epidemiology of infectious diseases as well as designing control and mitigation strategies [6,7].

Here, we consider how the rates of moving between compartments in standard epidemic models can be estimated from shedding data generated either when individuals are given an infectious dose of a virus under controlled conditions, or when existing cases are enrolled in a study, and the level of live virus that they produce (shed) is measured over time. The nature of these models and data means that there are not simple optimal values for the rate parameters, but instead the data constrain the parameters to lie close to a nonlinear curve in parameter space. We show that modern computationally intensive Bayesian methods that make use of information geometry can be used to calculate the posterior density for models of influenza, norovirus and Ebola. We then use forward modelling based on this posterior knowledge to show that some epidemiological conclusions are robust under the remaining uncertainty, but others require additional information to determine. In particular, for influenza, we show that the predicted effectiveness of quarantine-type interventions is unaffected by the remaining uncertainty, but antiviral-like interventions have a bimodal uncertainty structure. For norovirus, we show that the frequency of epidemics is predictable under the remaining uncertainty, but the severity and timing of each seasonal epidemic is not. For Ebola, we are able to distinguish between high- and low-viraemic infectious disease progression, giving results that are consistent with population-level observations of the case fatality ratio.

## 2. Methods

### 2.1. Overview

Our methodological approach involves three related components. We start by defining the different compartmental disease models that we use in §2.2. These are defined in terms of each case's *natural history*, which we represent mathematically as a continuous-time Markov chain. We also show how these models can be used to make population-level predictions by assuming, for example, that an infectious individual will cause new cases at a rate *β*.

Then in §2.3 we consider how the natural history parameters can be estimated from shedding data. An important distinction will be between the expected rate at which individuals infect in the population (quantified using a rate like *β* above) and the measured intensity of shedding (quantified using log titre). We will generally assume a simple linear relationship between these using a scaling parameter that we call *τ*.

Finally in §2.4, we present the Bayesian approach to uncertainty quantification as well as the algorithms necessary to implement this for the complex posterior distributions that arise in the analysis of shedding data.

### 2.2. Compartmental models of infectious disease

In a compartmental approach, we model the state of an individual who has been infected with a pathogen at time *t* = 0 as an integer random variable *X*(*t*). We label the possible states after infection 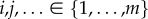; while more general structures are needed for other pathogens, for both influenza and norovirus we consider the ‘linear chain’ case where an individual spends an exponentially distributed period of time with mean 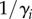 in each state *i* < *m* before progressing to state *i* + 1, and where *m* is the ‘recovered’ state, corresponding to the end of the infection, from which the individual does not move. In the electronic supplementary material, we provide a general solution for the probability of being in disease compartment *i* at time *t*,
2.1
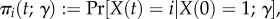
and its derivatives with respect to the rates 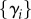. For Ebola, we consider a chain that branches, which introduces parameters relating to the probability of following one path or another in addition to rate constants.

We also assume that an individual has ‘infection rate’ *λ_i_* in state *i* (with *λ_m_* = 0), which is proportional to the rate at which they would infect new cases in a population-level epidemic model. A key quantity is the expected infectiousness of an individual over time
2.2
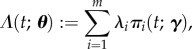
where the model parameters are 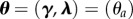. Perhaps the most important quantity in any epidemiological model of infections is the basic reproductive ratio, *R*_0_, defined as the expected number of secondary infections produced by a typical infectious individual early in the epidemic [4]. Under the simplifying (but frequently made) assumption of a homogeneous population, this quantity is given by
2.3
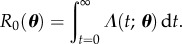
Note that the constants of proportionality ***λ*** depend on the nature and strength of interactions in the population and therefore cannot be determined from measurements of individuals alone.

#### 2.2.1. The SIR model

One of the simplest models in mathematical epidemiology is the SIR model, in which individuals are susceptible, infectious or removed. We use this model as a simple example of the methodology we propose. An individual infected at time *t* = 0 spends an exponentially distributed period of time in the infectious class, with rate *γ*, before recovering, and has infectiousness *β*. Therefore,
2.4
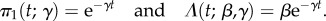
At the population level, supposing that we can ignore demographic processes such as births and deaths so the population size is fixed at *N*, we have a set of ordinary differential equations describing the evolution of an epidemic:
2.5
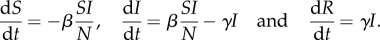
Here *S*(*t*) is the expected number of susceptible individuals in the population, *I*(*t*) is the expected number of infectious cases and *R*(*t*) is the expected number of removed individuals; we will use a similar notation below generalized in a natural way. In this work, we will consider how to fit expressions such as (2.4) to shedding data in such a way that population-level models such as (2.5) can be parametrized accounting for uncertainty.

#### 2.2.2. Influenza

Influenza is commonly modelled using the ‘SEEIIR’ or ‘SE_2_I_2_R’ framework, for example, in the work that was used to inform vaccination policy during the recent H1N1 pandemic [8]. Here *m* = 5 and individuals spend a 2-Erlang distributed period of time with mean 1/*ω* in a non-infectious ‘exposed’ state, and then a 2-Erlang distributed period of time with mean 1/*γ* in the infectious state, before recovering.

To show the possible different impacts of uncertainty in parameter values, we will consider two models for delayed intervention (implemented after a time period of length *d* after infection) during an influenza pandemic. The first of these assumes that the intervention is ‘quarantine-like’ and completely halts transmission after administration, leading to an epidemic with reproduction number
2.6
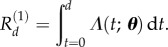
The second, however, assumes that the individual must not have progressed from the ‘latent’ to the ‘infectious’ class and is therefore similar to some models of administration of antiviral medication like Tamiflu [9,10], leading to an epidemic with reproduction number
2.7
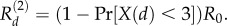


We note from standard results in mathematical epidemiology [4] that if the infection rate is *β*, and the mean infectious period is 1/*γ*, then the basic reproductive ratio is *R*_0_ = *β*/*γ*, and for an epidemic with reproduction number *R* the epidemic final size *Z* is given by the solution to the transcendental equation
2.8
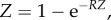
which we solve numerically for the two different reproduction numbers 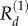 and 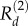 above, a range of delays, and fitted values of the parameters (*ω*, *γ*) at a fixed value of *R*_0_ = 1.4 (as in [8]) to make a direct comparison, although it would be straightforward to place a distribution on *R*_0_. We note that while all parameter values agree on the value of 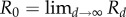, and that 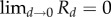, at finite non-zero *d* the uncertainty in parameters will lead to posterior variability in *R_d_* and hence *Z*.

Temporal features of an influenza epidemic are often more relevant for policy than the final size [11,12] and are typically considered using systems of differential equations. As we are considering an intervention with fixed delay, we couple the standard ODE system [8] to a set of terms modelling the delayed intervention leading to the delay-differential equation system
2.9
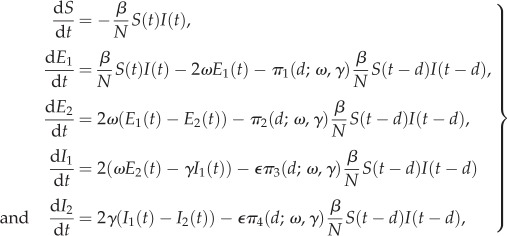
where the variable 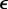 is 1 if the intervention works for any infected individual and 0 if it only works during the latent period, and *d* is the intervention delay. 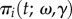 is the probability of being in state *i* time *t* after starting in state 1 at time 0 as in (2.1) above and is also determined by the parameters in the shedding model.

#### 2.2.3. Norovirus

Norovirus is usually assumed to follow the ‘SEIRS’ framework [13], where after infection an individual spends an exponentially distributed period of time with mean 1/*ω* in a ‘latent’ class, then an exponentially distributed period of time with mean 1/*γ* in the infectious class. In contrast to influenza, individuals move from the ‘recovered’ class back to the ‘susceptible’ class with rate *μ*; this loss of immunity is a relatively slow process that does not impact on the analysis of shedding data. It does, however, influence the population-level disease dynamics of norovirus, which can be described using a set of ordinary differential equations, where *S*(*t*) stands for the expected number of people who are susceptible and similarly for other compartments:
2.10
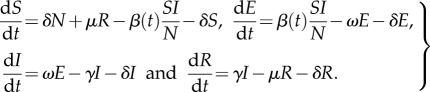
Here we have assumed a constant effective population size *N* and have a time-varying infection rate (which is necessary to reproduce the regular seasonality seen in real data [13]) that we assume takes a sinusoidal form 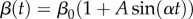. Because such external forcing in transmission is typically believed to arise from school terms [14], we take *A* = 1/3 to be close to existing empirical estimates of the impact of school closures on disease spreading [15,16], and *α* can be set to 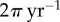. The demographic rate *δ* is standardly set to 1/70 yr^–1^. From the results of [17], we have that 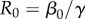; we vary this and the rate of waning immunity *μ* within ranges suggested by Simmons *et al*. [13], and then run the model (2.10) to determine its long-term behaviour for different fitted values of the parameters (*ω*, *γ*).

A norovirus vaccine is likely to be available in the future [18], and we model the impact of a vaccination policy starting at time *u* and with effective coverage *v* (defined as the product of coverage and efficacy) by modification of the demographic term for *S* and addition of a vaccinated *V* compartment:
2.11
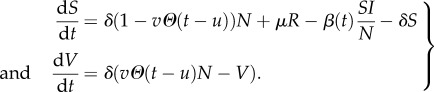
Here 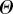 is the step function, leaving us with a set of time-inhomogeneous ordinary differential equations.

#### 2.2.4. Ebola

**Figure 1.**
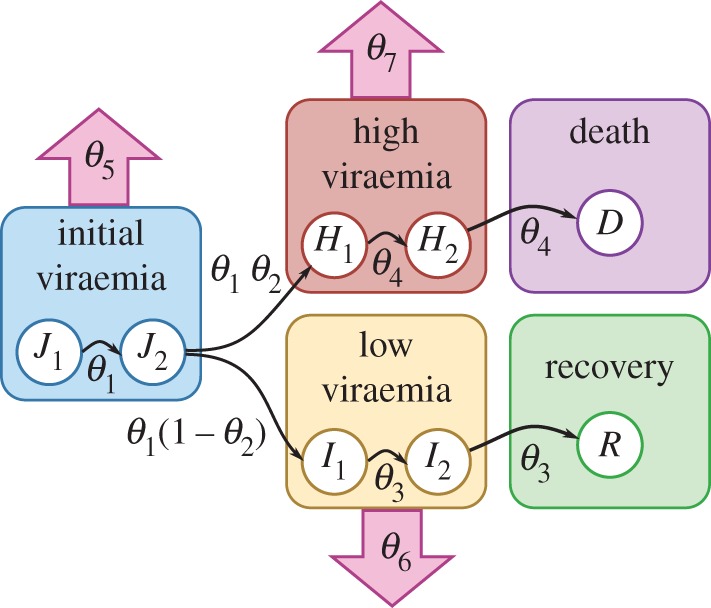
Ebola model compartmental structure and role of parameters. Compartments are shown as circles; flows between compartments are shown as thin black arrows labelled with parameters; and infectiousness is indicated by outward facing block arrows labelled with parameters.

Ebola is both much less common than influenza and norovirus and much more dangerous. This means that the modelling framework for it is less established—although it is typically assumed to follow an SEIR-type framework [19,20]—and also that challenge studies cannot be performed. Instead, existing cases are recruited and their viral loads are monitored. Our modelling approach is based on the results of such studies [21]. This is shown diagrammatically in figure 1 and is described by equations
2.12
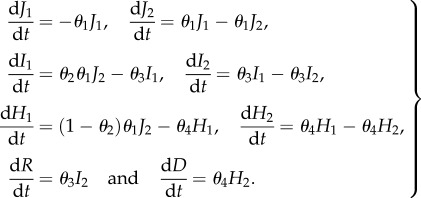
In this study design, the latent *E* states are not observed and so the initial condition is 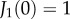 with all other quantities initially 0. Parameter interpretations and priors are given in table 1.

**Table 1.** Parameters of the Ebola model, their interpretation and prior distribution.

parameter	interpretation	prior
*θ*_1_	2/(mean time in initial viraemic state)	Exp(0.01)
*θ*_2_	proportion entering high-viraemic state	Uniform([0,1])
*θ*_3_	2/(mean time in low-viraemic state)	Exp(0.01)
*θ*_4_	2/(mean time in high-viraemic state)	Exp(0.01)
*θ*_5_	scaling parameter for initial viraemic state	Exp(0.1)
*θ*_6_	scaling parameter for low-viraemic state	Exp(0.1)
*θ*_7_	scaling parameter for high-viraemic state	Exp(0.1)

The transmission rates for low- and high-viraemic pathways are, respectively,
2.13
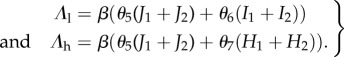


### 2.3. Shedding model and data

Our aim is to extract parameter estimates for compartmental epidemic models from challenge studies in which human volunteers are infected with a pathogen, or observational studies based on existing cases, and the level of live virus they produce (or ‘shed’) is measured over time. The concentration of live virus is quantified as a ‘titre’, which is essentially an estimate of the concentration of live virus. The relationship between this quantity and transmissibility is complex, but generally agreed to be sub-linear [22,23]. We assume here that at each time point *t* the log titre *y* is proportional to the expected intensity of infection *Λ*(*t*) plus additive Gaussian noise representing experimental error and other factors such as individual-level variability, leading to likelihood functions based on products of normal distribution probability mass functions. The details are, however, different for the four scenarios we consider and so we define our models separately below.

#### 2.3.1. The SIR model

For the SIR model, we assume one observation *y* of shedding with standard deviation *σ* at time *t* leading to likelihood function
2.14
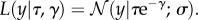
We will use this as a toy model to demonstrate our methodological approach, using numerical values *y* = 1, *σ* = 0.02 and *t* = 1. Note that here and throughout, we write the probability density function for the normal distribution with mean *μ* and standard deviation *σ* evaluated at *x* as
2.15
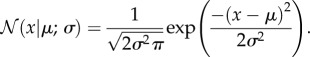


#### 2.3.2. Influenza

**Figure 2.**
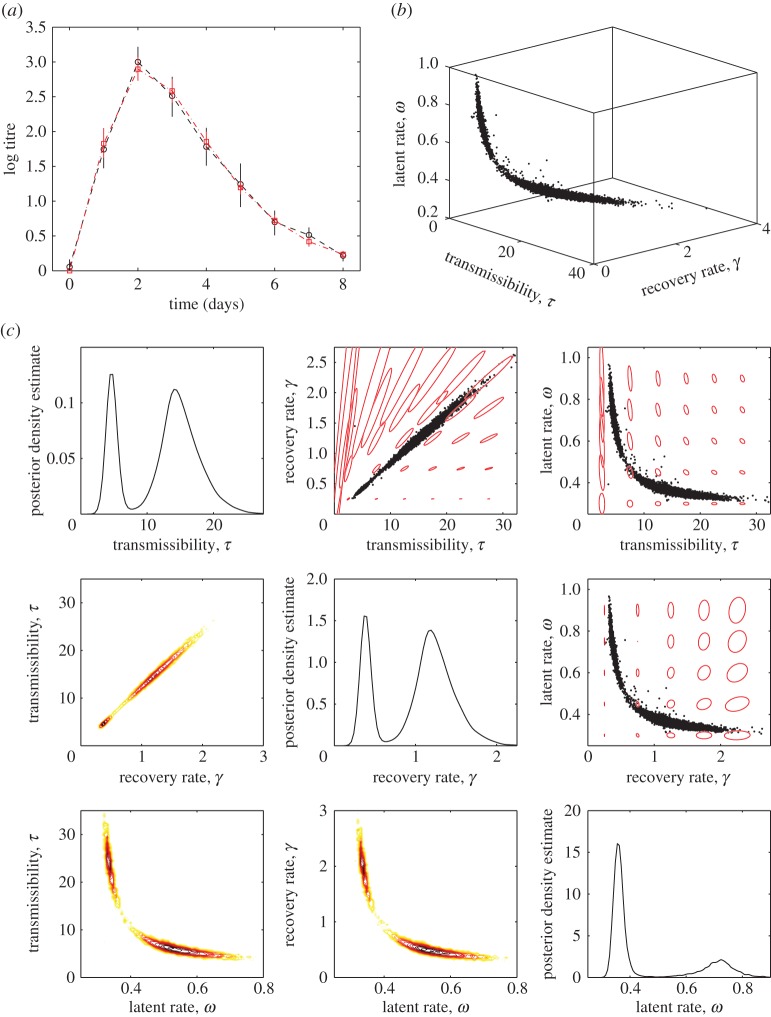
Analysis of influenza shedding data. (*a*) Data and empirical confidence intervals (black) against model mean and 95% credible interval (red). (*b*) Posterior samples obtained using WHLMC. (*c*) Diagonal plots: marginal posterior density estimates for each parameter. Bottom left plots: marginal log posterior for pairs of parameters—first-order gradients will be perpendicular to contours. Top right plots: samples (black) with visualizations of the metric—based on expectations of second-order derivatives as detailed in the main text—as red curves, centred on evenly spaced points, whose radius is inversely proportional to the infinitesimal distance at that angle as defined in (2.28).

For influenza, we use the meta-analysis data of Carrat *et al*. [24] for viral titre in the nasal passages of individuals infected with influenza A H1N1, which is shown in figure 2*a*. Here, observations are made at regularly spaced times belonging to a set 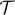. Under our general modelling assumptions, the likelihood of observing a set of mean log-titres (*y*_t_) among participants, with associated standard deviations (*σ*_t_), given the model parameters, is
2.16
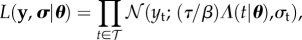
where 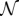 is the normal distribution probability density function intended to capture various sources of experimental error and individual-level variability as would be expected due to the central limit theorem. Here, we assume that the variance at each time point is measured, as given in [24]. Because the infection rate *β* depends *inter alia* on the rate of contact between individuals, which cannot be estimated from shedding data, we rescale the infectiousness *Λ* using the scaling parameter *τ* meaning that our parameters for estimation are 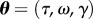.

#### 2.3.3. Norovirus

**Figure 3.**
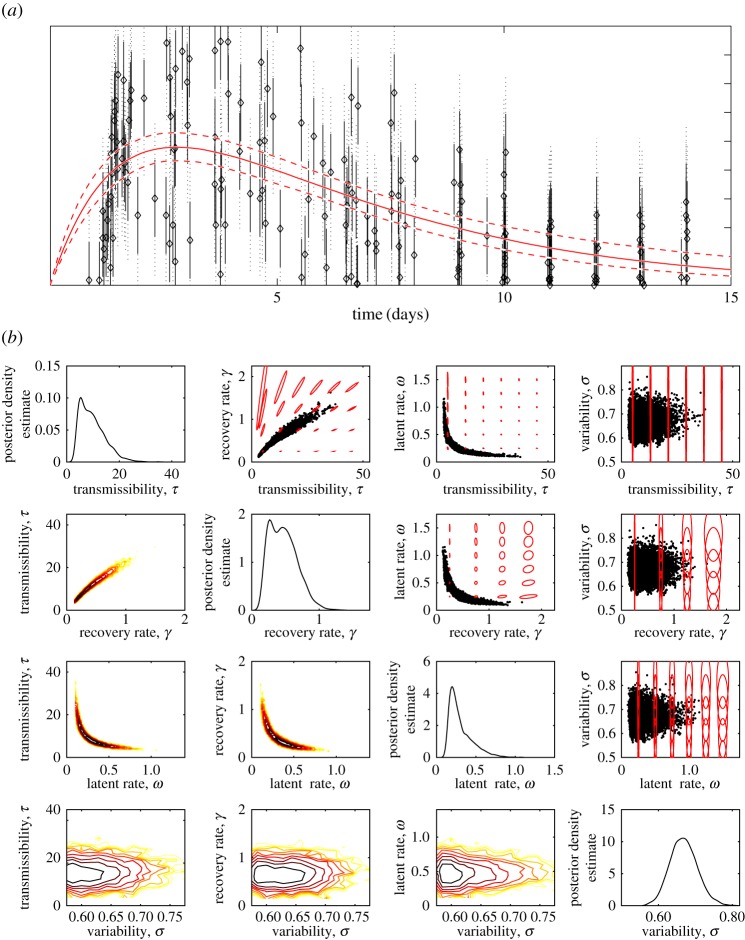
Analysis of norovirus shedding data. (*a*) Data (black circles) with 67% (solid black line) and 95% (dotted black line) against model mean and 95% credible interval (red). (*b*) Diagonal plots: marginal posterior density estimates for each parameter. Bottom left plots: marginal log posterior for pairs of parameters—first-order gradients will be perpendicular to contours. Top right plots: samples (black) with visualizations of the metric—based on the empirical Fisher information as detailed in the main text—as red curves, centred on evenly spaced points, whose radius is inversely proportional to the infinitesimal distance at that angle as defined in (2.28).

For norovirus, we use data from the study of Atmar *et al*. [25], where individuals were infected under controlled conditions and observations of viral titre in faeces made irregularly at times belonging to a set 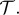 These data are shown in figure 3*a* and since they do not aggregate in the same way as the influenza data the likelihood function is
2.17
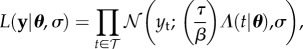
so *σ* is here an additional parameter that must be estimated, and we have rescaled the infectiousness as before using *τ*. This makes the norovirus parameter space dimension 4, in contrast to dimension 3 for influenza, with 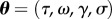.

#### 2.3.4. Ebola

For Ebola, we use the data of Ksiazek *et al*. [21] on viral titre in the blood of hospitalized Ebola cases, which are stratified into low- and high-viraemic disease pathways as shown in figure 1, together with the model described by equations (2.12). This leads to a likelihood function that takes the form of a product of low and high trajectories, each of which is similar to the influenza likelihood:
2.18
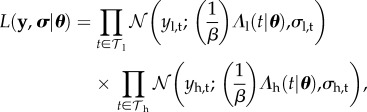
where we use a natural subscripting of ‘l’ for low and ‘h’ for high, and the parameters for estimation are 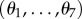 as shown in table 1.

### 2.4. Statistical framework

#### 2.4.1. The Bayesian approach to identifiability

It is long-established that fitting of a sum of exponentials to data is potentially troublesome; in particular, Acton [26] considers fitting the model 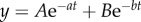 to (*y*, *t*) pairs and notes that ‘there are many combinations of (*a*, *b*, *A*, *B*) that will fit most exact data quite well indeed […] and when experimental noise is thrown into the pot, the entire operation becomes hopeless’.

Our compartmental models are more complex than sums of exponentials, but exhibit the same lack of a clear mode in the likelihood function. While there are various methods to address this issue in other applications (e.g. [27]), another response is (informally speaking) to consider all parameter combinations that fit well, and to investigate the epidemiological consequences of this uncertainty in parameter values.

More formally, we work in a Bayesian framework, meaning that we attempt to calculate the posterior density *p* over parameters ***θ*** from the likelihood function *L* and the prior function *f* using Bayes' rule
2.19
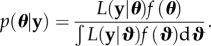
Given fixed data **y**, the measure 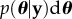 is higher in more credible regions of parameter space, and can be multi-modal and/or with many combinations of parameters having the same level of posterior support.

Here, we attempt to use priors that are broadly speaking uninformative—either uniform or improper if there is sufficient data, or low-rate exponential if there is less data. It is important to note, however, that use of strongly informative priors is another method for restoration of identifiability, in the sense of an approximately multivariate normal posterior distribution that is concentrated in the region of a unique mode.

#### 2.4.2. Markov chain Monte Carlo

Typically, the integral in the denominator of (2.19) is not tractable so we adopt the popular methodology of defining a Markov chain on parameter space whose stationary distribution has probability density function *p*, i.e. Markov chain Monte Carlo (MCMC) [28,29], in particular the Metropolis–Hastings algorithm [30,31]. This is a discrete-time Markov chain in which a change of state from 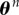 to 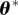 is proposed with probability 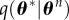 and the proposal is accepted with probability
2.20
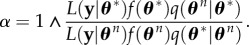
We shall now outline five popular approaches to MCMC, three that do not make use of derivatives and two that do, with all being in some sense a special case of (2.20).

#### 2.4.3. Derivative-free Markov chain Monte Carlo algorithms

(i) *Independence sampling.* In independence sampling, there is no dependence on the current state for the proposal distribution. A popular choice is simply to sample from the prior so that
2.21
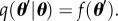
Intuitively, such an approach is expected to work well when the posterior is ‘close’ to the prior.

(ii) *Random walk*. In a random walk approach, the current state of the Markov chain is only used to inform the mean of the proposal distribution. The most popular choice is the multivariate normal
2.22
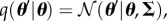
where the constant matrix **Σ** is often adaptively tuned to optimize algorithmic performance [32].

(iii) *Gibbs*. If it is possible to sample from the marginal posterior for a parameter 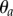 then we can propose with density
2.23
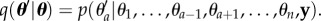
From (2.19) and (2.20), we then see that the acceptance probability for such a proposal is 1. If the marginal posteriors for all parameters are known, then pure Gibbs sampling can be undertaken and involves cycling through proposals (2.23) for all *a*.

#### 2.4.4. Derivative-based Markov chain Monte Carlo

In the field of numerical optimization, methods such as gradient descent that make use of the first-order derivatives of the function to be optimized are popular. Significant care must be taken when extending these to stochastic algorithms such as MCMC, but there are two popular methods that make use of the first-order derivatives of 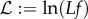. We use the following notation:
2.24
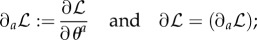
note that throughout we write (*x_i_*) for the vector **x** with *i*th element *x_i_* and (*M_ij_*) for the matrix **M** with (*i*, *j*)th element *M_ij_*. There are then two main families of derivative-based MCMC algorithms that we consider.

(i) *MALA*. The first algorithm family starts with the Langevin equation
2.25
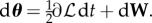
This stochastic differential equation model has a stationary distribution equal to the posterior distribution as defined in (2.19), and the Metropolis-adjusted Langevin algorithm (MALA) uses the Euler approximation to (2.25):
2.26
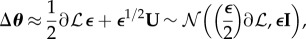
where **U** is a vector of independent standard normal random variables and **I** is the identity matrix. The approximation (2.26) can then be used as a proposal within the Metropolis–Hastings algorithm [33].

(ii) *HMC*. The second family of algorithms starts from the following system of ordinary differential equations that are a special case of Hamiltonian dynamics:
2.27
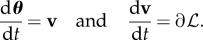
The randomness in the proposals arises as a result of the starting value of a vector of auxiliary variables **v**, by default chosen as a vector of standard normal random variables. MCMC algorithms based on Hamilton's equations (2.27) are called hybrid [34] or Hamiltonian [35] Monte Carlo (HMC).

One important thing to note about these algorithms is that they use likelihood derivatives to improve acceptance rates, but since they include a Metropolis–Hastings step the derivative calculations could be approximate.

#### 2.4.5. Geometric concepts in Markov chain Monte Carlo

While both MALA and HMC remove some of the inefficiencies of random walks through use of local gradients, they are not particularly efficient for the curved ‘boomerang’-shaped posteriors that we see for the shedding models and data defined above. To address this issue, recent work has made progress through use of concepts from differential geometry [36].

In general, we have *n* real-valued parameters, where the support for the posterior distribution is a set 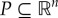. A general vector of parameter values is 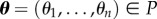, and informally speaking a metric is defined as a smooth symmetric map 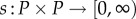 such that 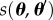 is the ‘distance’ between ***θ*** and ***θ***. In practice, we will only need to define this over small local distances, which requires a metric matrix 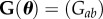; explicitly, the infinitesimal distance between ***θ*** and ***θ*** + d***θ*** is
2.28
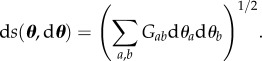
Here **G** can be any smooth matrix function and conceptually speaking this means that even the most complex posterior can be efficiently sampled with a choice of metric that brings all high-density regions of parameter space sufficiently ‘close’ to each other.

Throughout this work, we will visualize the impact of the metric in the plane for two parameters of a general model using ellipses, which are defined as follows. Let 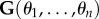 be the metric matrix and consider the first two parameters 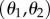 without loss of generality. Now let
2.29
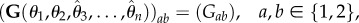
where 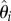 is a point estimate (we choose the posterior median) for the parameter *θ_i_*. Consider the ellipse defined by the following equation for polar coordinates distance *r* (from 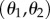 in the plane) and angle *α*:
2.30
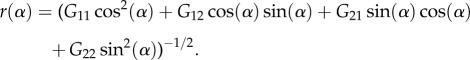
Plotting several such ellipses in the plane allows us to visualize the impact of the metric in the following sense: points on each ellipse are all the same ‘distance’ from the centre as each other under the assumption of a locally constant metric.

While it is not simple to optimize the metric for a particular model, a generally well-motivated choice is the Fisher–Rao metric as suggested by Girolami & Calderhead [36]
2.31
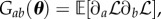
where the expectation is taken over data. Benefits of this metric include that it ensures the matrix **G** will be positive definite, and hence that the inverse matrix **G**^−1^ will exist and be positive definite. Calculations of the Fisher–Rao metric for the models under consideration are given in the electronic supplementary material, showing that it is also available in a closed form for our models. We note that other metrics are sometimes preferable, as discussed by, for example, Betancourt [37]; however in our case the Fisher–Rao metric proved to be adequate.

We used two different geometric algorithms, chosen based on features of the posterior.

(i) *SMMALA.* The simplified manifold Metropolis-adjusted Langevin algorithm (SMMALA) was introduced in [36] and shown to be competitive in terms of computational effort in several applied contexts by Calderhead and co-workers [38,39]. In this approach, the proposal distribution is
2.32
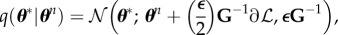
with standard MALA recovered if we set **G** = **I**. Note that the inverse of the metric matrix is used to ensure that the expected distance (as defined in (2.28)) of a move is directionally invariant. We used SMMALA to sample from the norovirus and Ebola posteriors, which each had one mode but were strongly correlated with variable local correlation structure.

(ii) *WHLMC*. The idea behind wormhole Lagrangian Monte Carlo (WHLMC) is that for a multi-modal posterior, a metric can be defined that dramatically reduces the distance between modes, and a modified form of the dynamics (2.27) can exploit this proximity. Full details of the algorithm are highly technical and are given in the papers that first introduced it [40,41], as well as in our electronic supplementary material.

## 3. Results

### 3.1. Selection of Markov chain Monte Carlo algorithm

**Figure 4.**
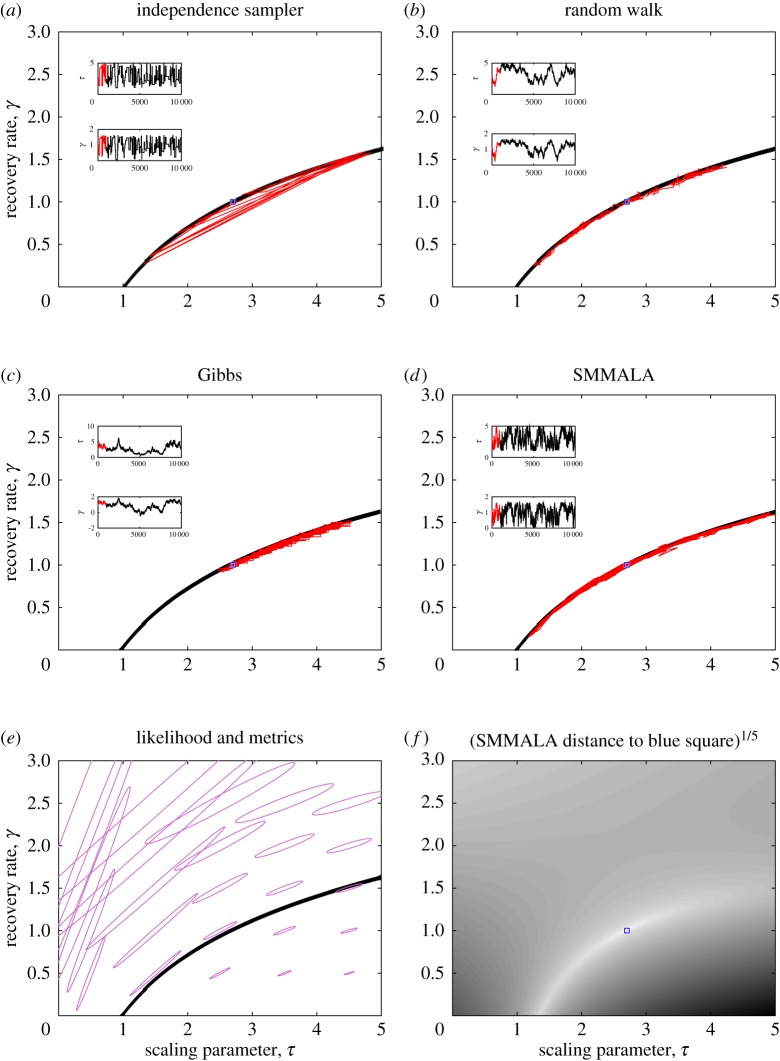
Results for the SIR model with recovery rate *γ* and scaling parameter *τ* (the constant of proportionality for the observed level of shedding at a given expected infectiousness). (*a*–*d*) MCMC trajectories for 1000 likelihood evaluations starting from the blue square are shown as red lines, with trace plots for a further 9000 steps shown in insets for different algorithms. (*e*) Visualizes the impact of the metric through the use of ellipses as detailed in §2.4.5. (*f*) Visualizes the impact of the metric through shading with shading intensity dependent on the distance to the starting point of the MCMC run.

Taking the simple likelihood function (2.14) together with prior distribution uniform ([0, 5] × [0, 3]) we were able to run the full set of MCMC algorithms discussed above to assess their efficiency. Figure 4 shows the results of running these algorithms.

In terms of the derivative-free algorithms, the low-dimensional nature of the problem means that the independence sampler does relatively well. Both of the random walk and Gibbs samplers are not able to move efficiently through the region of high posterior density due to its narrow, curved structure. By contrast, SMMALA is able to adjust to variations in local posterior structure and as such provides a series of samples from the posterior that are much more independent of each other than other approaches. This is explained by figure 4*e,f* that visualize the impact of the geometry in this algorithm.

**Figure 5.**
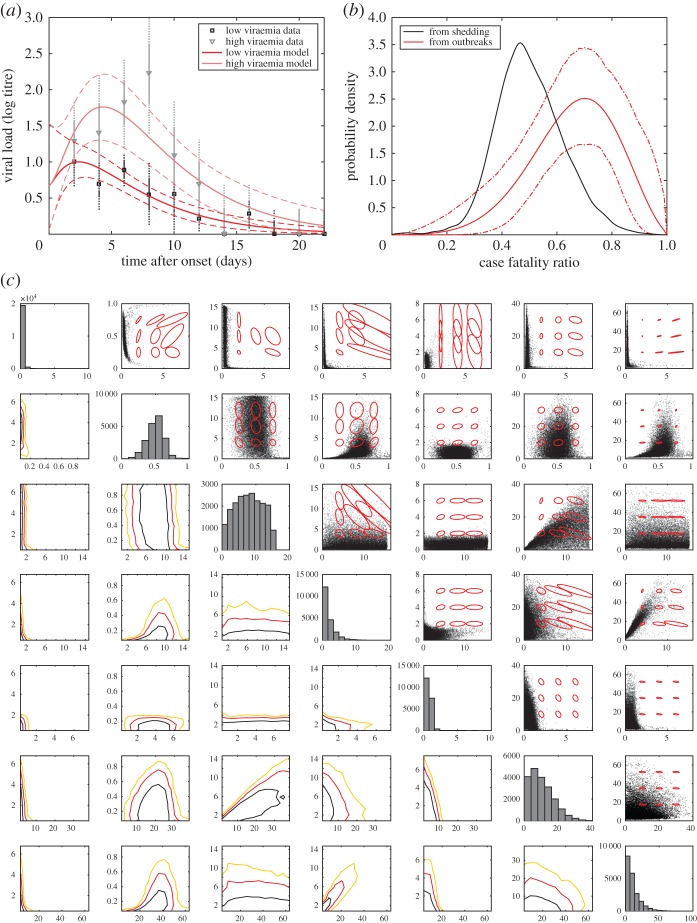
Ebola results. (*a*) Point estimate and posterior predictive interval for shedding over time against data for different viraemia levels. (*b*) Case fatality ratio from shedding data against that obtained for outbreaks [42]. (*c*) Posterior as for influenza and norovirus, with the *a*th row/column corresponding to parameter *θ_a_*.

Figures 2, 3 and 5 also show that the Fisher–Rao metric and associated geometry generally correctly resolves the difficulties associated with our highly correlated posterior distributions for influenza, norovirus and Ebola, allowing accurate quantification of uncertainty in epidemiological rates. The question then becomes under what circumstances the additional computational effort of implementation of these algorithms is warranted, which has no simple answer here as in other areas of computational statistics; however, we note the following points.

First, standard measures of performance such as minimum effective sample size per CPU second [36] often overstate the effectiveness of inaccurate algorithms for our models. Figure 4*b* shows that over iterations 2–3000, random walk sampling appears to be well-behaved and would yield a high ESS despite only being in a small sub-area of the region of parameter space from which we would like to sample.

Secondly, if the posterior density is concentrated in a nonlinear region, derivative-free methods such as Gibbs, independence sampling and random walk will have a general tendency to get ‘stuck’ in sub-areas. This will not be problematic if there are sufficient computational resources available to perform significant thinning—i.e. removal of MCMC samples to reduce correlations between those that remain.

Thirdly, computational resources for these algorithms will almost certainly become overstretched in any of the following three limits: (i) As the dimensionality of parameter space becomes larger, for example, in our Ebola model. (ii) In the presence of multi-modality as in our influenza model. (iii) For extreme cases of unidentifiability, for example, the *σ* → 0 limit of our SIR model.

Finally, the geometric methods for MCMC that we present and employ here are designed to be particularly well suited to complex nonlinear relationships between parameters where the derivatives of the log-likelihood and log-prior are available in an analytically closed form, which is the case for our models. Despite this we note that there are many other sophisticated approaches to computationally intensive Bayesian inference [29] that could be of use due to their generality.

### 3.2. Influenza parameters and antiviral treatment

Figure 2 shows the results for our influenza model given rate-0.1 exponential priors on each parameter (chosen not to influence the posterior significantly but to ensure that the small number of data points does not become problematic). This shows that the credible ranges of individual parameters are close to typical values in the literature—Baguelin *et al*. [8], for example, consider scenarios with 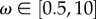 and 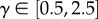.

**Figure 6.**
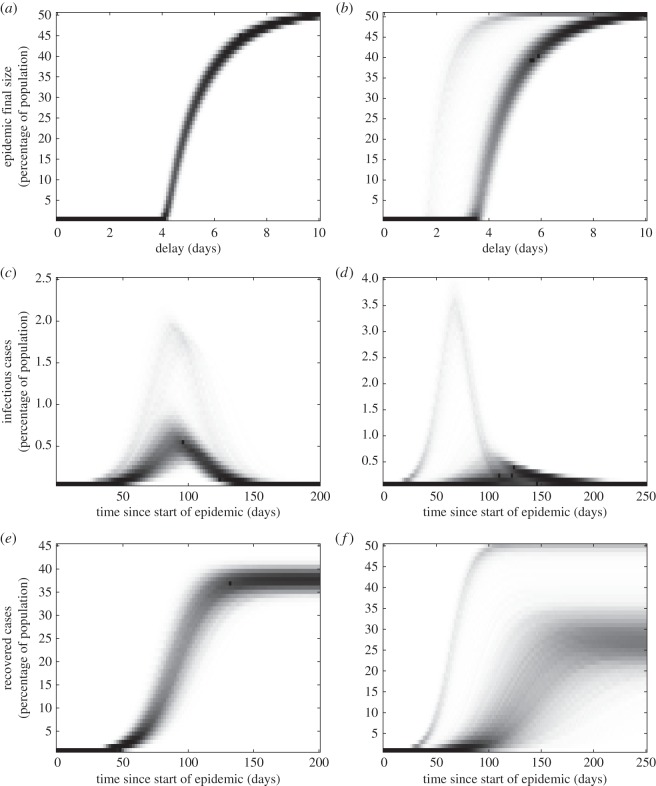
Interventions administered after a constant delay against influenza given parameter uncertainty. (*a*,*c*,*e*) Assuming that the intervention always stops transmission. (*b*,*d*,*f*) Assuming that the intervention only stops transmission if the individual has not yet become infectious. (*a,b*) Final sizes for a range of intervention delays. Plots of infection (*c,d*) and recovery (*e,f*) time series are shown for delay *d* = 6 (*c,e*) and *d* = 4.5 (*d,f*). Intensity of shading is proportional to probability^0.1^ to render features visible in regions with large uncertainty.

More importantly, however, the bimodal and highly correlated nature of the posterior distribution means that for some models of antiviral action it is not possible to make firm predictions based on parameter values from challenge studies. Figure 6*a,c,e* shows the results of a quarantine-like intervention that is always effective after a delay, where the relationship between delay in antiviral administration and epidemic final size at constant *R*_0_ is predictable to within a few percentage points, although there is much greater uncertainty in the peak prevalence. Figure 6*b,d,f* shows an antiviral-like intervention that is only effective if administered during the latent period, meaning the absolute uncertainty in final size can be almost 50% and the relative uncertainty in peak prevalence can amount to a factor of four or more.

### 3.3. Norovirus parameters and seasonality

Figure 3 shows the results for our norovirus model; given the large amount of data we use improper priors and see that the credible ranges of individual norovirus parameters are also close to typical values in the literature; e.g. Simmons *et al*. [13] take *ω* = 1 and *γ* = 0.5.

**Figure 7.**
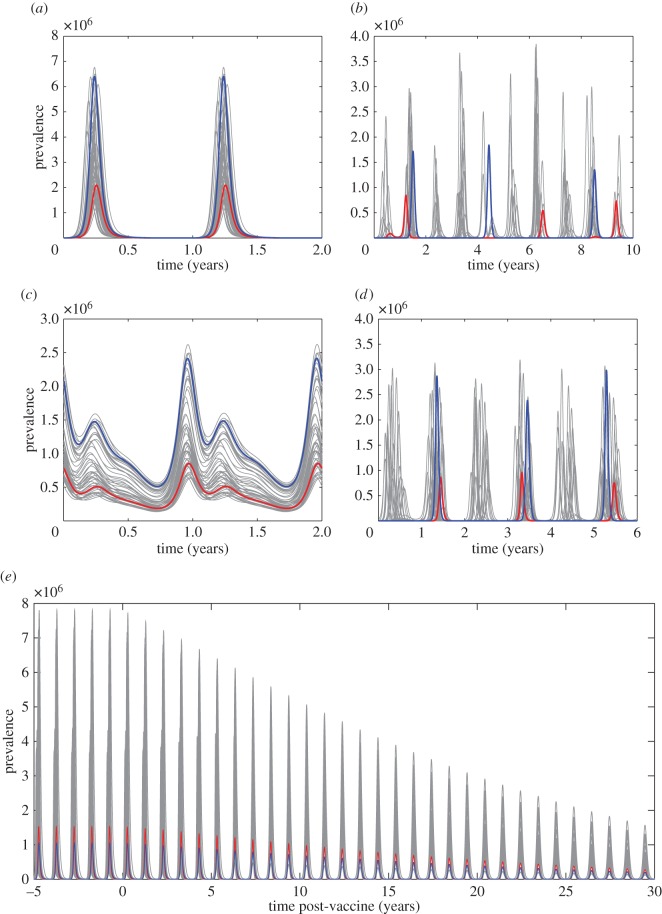
Implications of uncertainty in norovirus shedding for long-term temporal behaviour. Deterministic ODE trajectories are shown for 50 different posterior samples with two randomly chosen ones highlighted in red and blue. (*a*) *R*_0_ = 1.8, *T_L_* = 6 months shows regular annual oscillations. (*b*) *R*_0_ = 1.8, *T_L_* = 60 months shows irregular/chaotic behaviour. (*c*) *R*_0_ = 4, *T_L_* = 6 months shows annual oscillation but not well-defined epidemics. (*d*) *R*_0_ = 4, *T_L_* = 60 months shows bi-annual oscillation but with variable peak heights. (*e*) Time series for parameters as in plot (*a*) given vaccination at birth with 90% efficacy.

Figure 7 shows that the impact of this uncertainty (for other parameter values as given above) is mainly seen in the height (with peak prevalence differing by a factor of 3 or more) and timing within the year of seasonal epidemics. For the chaotic/irregular scenario (figure 7*b*) however, the overall epidemic dynamics are subject to significant uncertainty. Fortunately, conditioned on knowing that epidemics are regular and annual and with a particular peak, the broad impact of a vaccine policy can be predicted as shown in figure 7*e*.

### 3.4. Ebola parameters and case fatality ratio

Figure 5 shows the results obtained for our Ebola model. The point estimates and 95% CIs of mean infections period come out at 5.3[3.3, 7.4] days for low-viraemia cases and 6.8[5.1, 10.1] days for high-viraemia cases, which are reasonable values [20]. Figure 5*a* shows that the model produces shedding output that is consistent with the data, and figure 5*b* compares the posterior for the case fatality ratio obtained from shedding data to the one obtained from known outcomes of previous outbreaks [42], again suggesting that the model outcome is reasonable but that uncertainties are very large.

## 4. Discussion

In summary, we have shown that it is possible to use modern Bayesian MCMC methods, based on derivatives of the log-likelihood and information geometry, to make a full uncertainty quantification of epidemiological parameters fitted to human viral shedding data. We have performed our analysis for two of the most prevalent pathogens: influenza and norovirus, as well as for Ebola, a highly virulent zoonotic disease.

Shedding data allow disease ‘natural history’ parameters to be fitted; these usually need to be combined with population-level measurements such as the basic reproductive ratio *R*_0_ to specify policy-relevant models fully. Our results show that the epidemiological consequences of uncertainty in natural history parameters can often be highly significant since these are important for interventions such as reducing transmission through quarantine or medication, as well as prediction of long-term disease behaviour and clinical outcomes. Natural history parameters also strongly affect other aspects of infectious disease epidemiology such as outbreak reconstruction and we would expect similarly strong effects in these contexts.

To make progress, we have had to base our analysis on simplifying assumptions that we would hope can be relaxed in future work as the field develops. One example is that the simple likelihood functions (2.18) and (2.17) assume independence that could be extended to include more general functional relationships, which would be particularly important if the methodology were extended to diseases such as human immunodeficiency virus where there are very different time scales involved in passing between compartments [43]. In such an example, one might wish to consider more general models, for example, ones in which the progression between the latent, infectious and removed classes is governed by more general distributions than those we have considered here. Provided the Laplace transformations of the probability density functions for these distributions are available, then expressions for *Λ* and its derivatives with respect to the parameters can be obtained via the convolution theorem, although this can result in a computationally intensive likelihood function. Alternatively, it might be possible to approximate the derivatives since inaccuracies in any such approximation will lead to algorithmic inefficiency rather than bias.

An additional assumption we have made is that the parameters we are not fitting (for example, the basic reproductive ratio *R*_0_) are fixed. This is particularly important to relax if multiple data sources are to be used in a principled way in infectious disease modelling for public health [2]. In particular, the measurements at the population level required to estimate *R*_0_ are likely to carry their own uncertainty, which can be combined with our uncertainty quantification for disease natural history parameters as the next step towards systematic evidence synthesis for infectious diseases.

## Supplementary Material

Supplementary Material
