# Supplementary material for: Bayesian uncertainty quantification for transmissibility of influenza, norovirus and Ebola using information geometry
Source: J R Soc Interface. 2016 Aug;13(121):20160279. doi: 10.1098/rsif.2016.0279 (PMC5014059; doi:10.1098/rsif.2016.0279)
Supplement: Supplementary Material [file rsif20160279supp1.pdf]

Bayesian uncertainty quantification for transmissibility of influenza, norovirus and Ebola using information geometry: Supplementary Material

Thomas House Ashley Ford Shiwei Lan Samuel Bilson
Elizabeth Buckingham-Jeffery Mark Girolami

Here we present technical and mathematical results supporting the main paper.

Contents

Contents **1**

1 Solution of the linear compartmental model **2**
1.1 Model definition . . . 2
1.2 Distinct rates . . . 2
1.2.1 Solution via eigenvalue decomposition . . . 2
1.2.2 Solution via Laplace transform . . . 4
1.2.3 Derivatives . . . 5
1.3 Arbitrary rates . . . 6
1.3.1 Solution . . . 7
1.3.2 Derivatives . . . 9

2 Metric for the shedding models **11**
2.1 Underlying Fisher-Rao metric . . . 11
2.2 The SIR model . . . 12
2.3 Influenza and Ebola . . . 12
2.4 Norovirus . . . 12
2.5 Other contributions to the metric . . . 13

3 The WLMC algorithm **13**
3.1 Lagrangian Monte Carlo (LMC) . . . 13
3.2 WLMC . . . 14
3.3 Wormhole Metric . . . 14
3.4 Wormhole Network . . . 14

4 Supplementary figure **16**

5 Mathematica code **16**

References **17**

1 Solution of the linear compartmental model

1.1 Model definition

Consider the continuous-time Markov chain $X = (X(t)|t \in \mathbb{R}^+)$ with state space $\mathcal{S} = \{i\}_{i=1}^m$, $m \in \mathbb{N}$, and rates $\boldsymbol{\gamma} = (\gamma_1, \dots, \gamma_{m-1})$, $\gamma_i \in \mathbb{R}^+, \forall\, i \in \mathcal{S} \setminus \{m\}$. For convenience we will set $\gamma_m = 0$.

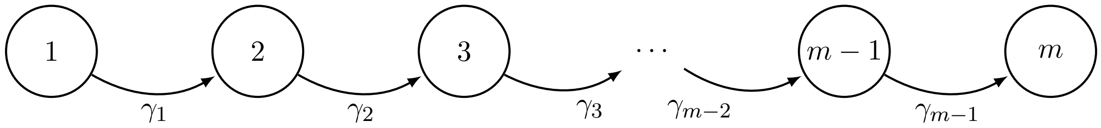

This has the following $m \times m$ generator matrix

$$\mathbf{M} = \begin{pmatrix} -\gamma_1 & & & & \\ \gamma_1 & -\gamma_2 & & 0 & \\ & \gamma_2 & \ddots & & \\ & & \ddots & -\gamma_{m-1} & \\ & 0 & & \gamma_{m-1} & 0 \end{pmatrix}, \tag{1}$$

which in component form reads

$$M_{ij} = \begin{cases} \gamma_j(\delta_{i-1,j} - \delta_{ij}) & \text{if } j \neq m\ , \\ 0 & \text{otherwise.} \end{cases} \tag{2}$$

X has an absorbing state at $i = m$, a unique stationary distribution π^* such that $[\pi^*]_i = \delta_{i,m}$, but it is not irreducible, ergodic or reversible (since $p_{ij}(t) = 0, \forall\, i \leq j \in \mathcal{S}$).

We now try to solve the system

$$\frac{\mathrm{d}\boldsymbol{\pi}}{\mathrm{d}t} = \mathbf{M}\boldsymbol{\pi}, \quad \pi_i(0) = \delta_{i1}\ , \tag{3}$$

for different relationships between the rates γ.

1.2 Distinct rates

The solution to (3) is simpler if all of the rates are distinct, i.e. if $\gamma_i \neq \gamma_j\, \forall\, i \neq j \in \mathcal{S} \setminus \{m\}$. We will use two different methods to solve this system.

1.2.1 Solution via eigenvalue decomposition

Here we start by finding the eigenvalues of $\mathbf{M}$, we solve the characteristic equation

$$\det(\mathbf{M} - \lambda \mathbb{1}) = 0 \tag{4}$$

By noting that the matrix $\mathbf{M} - \lambda\mathbb{1}$ is lower triangular, we see that its determinant is the product of its diagonal entries and so

$$-\lambda \prod_{i=1}^{N} (-\gamma_i - \lambda) = 0, \quad \Longrightarrow \quad \lambda = 0, -\gamma_1, -\gamma_2, \dots, -\gamma_{m-1}. \tag{5}$$

The eigenvectors equation can then be written in component form as

$$\sum_{k=1}^{i} M_{ik}[v_j]_k = -\gamma_j [v_j]_i \ , \quad \forall\, i,j \in \mathcal{S} \ . \tag{6}$$

Now define a matrix $\mathbf{A}$ whose i-th column is the i-th right eigenvector of $\mathbf{M}$ so that $\mathbf{M} = \mathbf{A}\mathbf{\Lambda}\mathbf{A}^{-1}$, where $\mathbf{\Lambda}$ is a diagonal matrix with i-th diagonal element equal to γ_i. Substituting for M_{ij} as in (2) and rearranging, we have

$$(\gamma_i - \gamma_j)A_{ij} = \gamma_{i-1}A_{i-1,j} \tag{7}$$

and so since $\gamma_{i-1} \neq 0$, $A_{ij} = 0$ for $i < j$. If we set $A_{ii} = 1$, then by induction we have

$$A_{ij} = [v_j]_i = \begin{cases} \displaystyle\prod_{k=j}^{i-1} \frac{\gamma_k}{\gamma_{k+1} - \gamma_j} & i > j \\ 1 & \text{if } i = j \ , \\ 0 & \text{otherwise.} \end{cases} \tag{8}$$

and so the matrix $\mathbf{A}$ is also lower triangular. We then write the solution to (3) as

$$\boldsymbol{\pi}(t) = \mathrm{e}^{\mathbf{M}t}\boldsymbol{\pi}(0) = \mathbf{A}\mathrm{e}^{\mathbf{\Lambda}t}\mathbf{A}^{-1}\boldsymbol{\pi}(0) = \mathbf{A}\mathrm{e}^{\mathbf{\Lambda}t}\mathbf{c} \ , \tag{9}$$

where $\mathbf{c}$ is a vector obeying $\mathbf{A}\mathbf{c} = \boldsymbol{\pi}(0)$. Clearly, $c_1 = 1$ and for $i \neq 1$

$$\sum_{j=1}^{i-1} c_j \prod_{k=j}^{i-1} \frac{\gamma_k}{\gamma_{k+1} - \gamma_j} + c_i = 0 \ . \tag{10}$$

Let us consider the possible solution

$$c_i = \begin{cases} 1 & \text{for } i = 1 \ , \\ \displaystyle\prod_{j=1}^{i-1} \frac{\gamma_j}{\gamma_j - \gamma_i} & \text{for } i \neq 1 \ . \end{cases} \tag{11}$$

Then for $i \neq 1$, (10) becomes

$$\begin{aligned} &\sum_{j=1}^{i-1} \prod_{k=1}^{j-1} \frac{\gamma_k}{\gamma_k - \gamma_j} \prod_{k=j}^{i-1} \frac{\gamma_k}{\gamma_{k+1} - \gamma_j} + \prod_{j=1}^{i-1} \frac{\gamma_j}{\gamma_j - \gamma_i} = 0 \\ \Longrightarrow \quad &\left(\sum_{j=1}^{i-1} \prod_{\substack{k=1 \\ k\neq j}}^{i} \frac{1}{\gamma_k - \gamma_j} + \prod_{k=1}^{i-1} \frac{1}{\gamma_k - \gamma_i} \right) \prod_{j=1}^{i-1} \gamma_j = 0 \end{aligned} \tag{12}$$

and so

$$\sum_{j=1}^{i} \prod_{\substack{k=1 \\ k\neq j}}^{i} \frac{1}{\gamma_k - \gamma_j} = 0, \ \text{since } \gamma_i \neq 0, \forall\, i \in \mathcal{S} \setminus \{m\}. \tag{13}$$

Now we can show that expression (13) holds for all $\gamma_i \neq \gamma_j \in \mathbb{R}, \ i \neq j$.

Proof. Let there be a set $\{\gamma_i\}_{i=1}^{n}$ s.t. $\gamma_i \in \mathbb{C}, \ n \in \mathbb{N}$ and $\gamma_i \neq \gamma_j \, \forall\, i \neq j$. Now consider the expression

$$P_i(x) = \prod_{\substack{j=1 \\ j\neq i}}^{n} (\gamma_j - x)$$

Using the formula for partial fraction decomposition

$$\frac{1}{P_i(x)} = \sum_{\substack{j=1 \\ j\neq i}}^{n} \frac{1}{P_i'(\gamma_j)} \frac{1}{x-\gamma_j}$$

we have that

$$\begin{aligned}
\prod_{\substack{j=1 \\ j\neq i}}^{n} \frac{1}{\gamma_j - \gamma_i} &= \sum_{\substack{j=1 \\ j\neq i}}^{n} \frac{1}{P_i'(\gamma_j)} \frac{1}{\gamma_i - \gamma_j} \\
&= -\sum_{\substack{j=1 \\ j\neq i}}^{n} \frac{1}{\gamma_i - \gamma_j} \prod_{\substack{k=1 \\ k\neq i,j}}^{n} \frac{1}{\gamma_k - \gamma_j} \\
&= -\sum_{\substack{j=1 \\ j\neq i}}^{n} \prod_{\substack{k=1 \\ k\neq j}}^{n} \frac{1}{\gamma_k - \gamma_j} \\
\implies \sum_{j=1}^{n} \prod_{\substack{k=1 \\ k\neq j}}^{n} \frac{1}{\gamma_k - \gamma_j} &= 0 \quad \square
\end{aligned}$$

Combining this with the eigenvectors/values of $\mathbf{M}$ we can substitute into (9) to give

$$\begin{aligned}
\pi_i(t) &= \sum_{j=1}^{i} A_{ij} c_j \mathrm{e}^{-\gamma_j t} \\
&= \begin{cases} \mathrm{e}^{-\gamma_1 t} & \text{for } i = 1 \ , \\ \prod_{k=1}^{i-1} \gamma_k \times \sum_{j=1}^{i} \mathrm{e}^{-\gamma_j t} \prod_{\substack{k=1 \\ k\neq j}}^{i} \frac{1}{\gamma_k - \gamma_j} & \text{for } i \neq 1 \ . \end{cases}
\end{aligned} \tag{14}$$

1.2.2 Solution via Laplace transform

For the more general case, there are benefits to a Laplace transform approach, which we will introduce here. We define the Laplace transform of a function $f : \mathbb{R}^+ \to \mathbb{R}^d$, $t \mapsto f(t)$ as $\mathscr{L}\{f\} : \mathbb{C} \to \mathbb{R}^d$, $s \mapsto \tilde{f}(s)$ such that

$$\hat{\mathscr{L}}\{f\}(s) := \tilde{f}(s) = \int_0^\infty f(t)\mathrm{e}^{-st}\mathrm{d}t \tag{15}$$

Laplace transforming (3) then gives

$$s\tilde{\boldsymbol{\pi}} - \boldsymbol{\pi}(0) = \mathbf{Q}\tilde{\boldsymbol{\pi}} \ . \tag{16}$$

If s is not an eigenvalue of $\mathbf{Q}$, then the matrix $s\mathbb{1} - \mathbf{Q}$ is invertible and

$$\tilde{\boldsymbol{\pi}} = (s\mathbb{1} - \mathbf{Q})^{-1}\boldsymbol{\pi}(0) \ , \quad \text{so} \quad \boldsymbol{\pi}(t) = \hat{\mathscr{L}}^{-1}\{(s\mathbb{1} - \mathbf{Q})^{-1}\boldsymbol{\pi}(0)\}(t) \ . \tag{17}$$

We can then calculate the inverse Laplace transform using the residue theorem. Considering the explicit form of (17) for our model, if we let $\mathbf{B}(s) = s\mathbb{1} - \mathbf{Q}$, where $s \neq -\gamma_i \, \forall \, i \in \mathcal{S}$, then $\mathbf{B}$ is invertible and we need to find $\mathbf{B}^{-1}\boldsymbol{\pi}(0)$ where $[\pi(0)]_i = \delta_{i1}$, or in component form

$$[\mathbf{B}^{-1}\boldsymbol{\pi}(0)]_i = \sum_{j=1}^{m} B_{ij}^{-1}\pi_j(0) = B_{i1}^{-1} \tag{18}$$

Using $\mathbf{B}\mathbf{B}^{-1} = \mathbb{1}$, and noting that $\mathbf{B}$ is lower triangular, we have that

$$\sum_{j=1}^{i} B_{ij} B^{-1}_{j1} = \delta_{i1} \tag{19}$$

For $i = 1$: $B^{-1}_{11} = 1/B_{11} = (s+\gamma_1)^{-1}$. For $i \neq 1$:

$$\begin{aligned}
&\sum_{j=1}^{i} B_{ij} B^{-1}_{j1} = 0 \\
&\implies \sum_{j=1}^{i} \left(-\gamma_j \delta_{i-1,j} + (s+\gamma_j)\delta_{ij}\right) B^{-1}_{j1} = 0 \\
&\implies B^{-1}_{i1} = \frac{\gamma_{i-1}}{s+\gamma_i} B^{-1}_{i-1,1} = \prod_{k=2}^{i} \frac{\gamma_{k-1}}{s+\gamma_k} B^{-1}_{11} \ ,
\end{aligned}$$

and so

$$[(s\mathbb{1} - \mathbf{Q})^{-1} \boldsymbol{\pi}(0)]_i = \begin{cases} \dfrac{1}{s+\gamma_1} & i = 1 \ , \\ \displaystyle\prod_{k=1}^{i-1} \gamma_k \times \prod_{k=1}^{i} \frac{1}{s+\gamma_k} & i \neq 1 \ . \end{cases} \tag{20}$$

Then finding $\pi_i(t)$ reduces to calculating $\hat{\mathscr{L}}^{-1}\{\tilde{f}_i(s)\}$ where $\tilde{f}_i(s) = \prod_{k=1}^{i} \frac{1}{s+\gamma_k}$. Since $\gamma_i \neq \gamma_j \, \forall \, i \neq j$, all the poles $s = -\gamma_i$ of $\tilde{f}$ are order 1, and so using the residue theorem we have

$$\begin{aligned}
\hat{\mathscr{L}}\{\tilde{f}_i(s)\}(t) &= \sum_{j=1}^{i} \mathrm{Res}[\tilde{f}_i(s)\mathrm{e}^{st}, -\gamma_j] \\
&= \sum_{j=1}^{i} \lim_{s \to -\gamma_j} (s+\gamma_j)\mathrm{e}^{st} \prod_{k=1}^{i} \frac{1}{s+\gamma_k} \\
&= \sum_{j=1}^{i} \mathrm{e}^{-\gamma_j t} \prod_{\substack{k=1 \\ k \neq j}}^{i} \frac{1}{\gamma_k - \gamma_j}, \ i \neq 1 \quad \text{or} \quad \mathrm{e}^{-\gamma_1 t}, \ i = 1 \ ,
\end{aligned} \tag{21}$$

and so finally

$$\pi_i(t) = \hat{\mathscr{L}}^{-1}\{[(s\mathbb{1} - \mathbf{Q})^{-1} \boldsymbol{\pi}(0)]_i\} = \begin{cases} \mathrm{e}^{-\gamma_1 t} & i = 1 \ , \\ \displaystyle\prod_{k=1}^{i-1} \gamma_k \times \sum_{j=1}^{i} \mathrm{e}^{-\gamma_j t} \prod_{\substack{k=1 \\ k \neq j}}^{i} \frac{1}{\gamma_k - \gamma_j} & i \neq 1 \ , \end{cases} \tag{22}$$

which is equivalent to (14).

1.2.3 Derivatives

Lets now take derivatives with respect to the model parameters $\{\gamma_i\}$. This can be most easily done in the Laplace domain since we can use the fact that

$$\partial_i \hat{\mathscr{L}}\{f\}(s) = \hat{\mathscr{L}}\{\partial_i f\}(s) \ , \text{ where } \quad \partial_i := \partial/\partial\gamma_i \ , \tag{23}$$

and so

$$\partial_i f(t) = \hat{\mathscr{L}}^{-1}\{\partial_i \hat{\mathscr{L}}\{f\}\}(t) \ . \tag{24}$$

If we let $\tilde{\boldsymbol{\pi}}_i(s) = [(s\mathbb{1} - \mathbf{Q})^{-1}\boldsymbol{\pi}(0)]_i$ then

$$\partial_i \tilde{\pi}_j(s) = \begin{cases} \dfrac{\tilde{\pi}_j(s)}{\gamma_i} - \dfrac{\tilde{\pi}_j(s)}{s+\gamma_i} & i < j \ , \\ -\dfrac{\tilde{\pi}_i(s)}{s+\gamma_i} & i = j \ , \\ 0 & i > j \ . \end{cases} \tag{25}$$

Using the convolution theorem for Laplace transforms, we have

$$\begin{aligned} \hat{\mathscr{L}}^{-1}\left\{\frac{\tilde{\pi}_j(s)}{\gamma_i} - \frac{\tilde{\pi}_j(s)}{s+\gamma_i}\right\} &= \frac{\pi_j(t)}{\gamma_i} - \int_0^t \pi_j(u)\mathrm{e}^{-\gamma_i(t-u)}\mathrm{d}u \\ &= \prod_{k=1}^{j-1}\gamma_k \times \left\{\sum_{\substack{n=1 \\ n\neq i}}^{j} \frac{\gamma_i \mathrm{e}^{-\gamma_i t} - \gamma_n \mathrm{e}^{-\gamma_n t}}{\gamma_i(\gamma_i-\gamma_n)} \prod_{\substack{k=1 \\ k\neq n}}^{j} \frac{1}{\gamma_k - \gamma_n} + \frac{1-\gamma_i t}{\gamma_i}\mathrm{e}^{-\gamma_i t}\prod_{\substack{k=1 \\ k\neq i}}^{j}\frac{1}{\gamma_k-\gamma_i}\right\} \\ &= \prod_{k=1}^{j-1}\gamma_k \times \sum_{\substack{n=1 \\ n\neq i}}^{j}\left(\frac{\gamma_n(\mathrm{e}^{-\gamma_i t} - \mathrm{e}^{-\gamma_n t})}{\gamma_i(\gamma_i-\gamma_n)} + t\mathrm{e}^{-\gamma_i t}\right)\prod_{\substack{k=1 \\ k\neq n}}^{j}\frac{1}{\gamma_k-\gamma_n} \end{aligned}$$

where we used the relationship proved in §1.2.1. Thus

$$\partial_i \pi_j(t) = \begin{cases} -t\mathrm{e}^{-\gamma_1 t} & i = j = 1 \ , \\ \displaystyle\prod_{k=1}^{i-1}\gamma_k \times \sum_{n=1}^{i-1}\left(\frac{\mathrm{e}^{-\gamma_i t} - \mathrm{e}^{-\gamma_n t}}{\gamma_i - \gamma_n} + t\mathrm{e}^{-\gamma_i t}\right)\prod_{\substack{k=1 \\ k\neq n}}^{i}\frac{1}{\gamma_k-\gamma_n} & i = j \neq 1 \ , \\ \displaystyle\prod_{k=1}^{j-1}\gamma_k \times \sum_{\substack{n=1 \\ n\neq i}}^{j}\left(\frac{\gamma_n(\mathrm{e}^{-\gamma_i t} - \mathrm{e}^{-\gamma_n t})}{\gamma_i(\gamma_i-\gamma_n)} + t\mathrm{e}^{-\gamma_i t}\right)\prod_{\substack{k=1 \\ k\neq n}}^{j}\frac{1}{\gamma_k-\gamma_n} & i < j \ , \\ 0 & i > j \ , \end{cases} \tag{26}$$

which can be checked by computing the derivative directly from (14).

1.3 Arbitrary rates

Consider a general, pure birth chain with $m = N+1$ states $\{I\}_{I=1}^{N+1}$, and M distinct parameters $\{\gamma_i\}_{i=1}^{M}$, defined by the following $(N+1)\times(N+1)$ generator matrix $\mathbf{Q}$, where $\gamma_i \neq \gamma_j \in \mathbb{R}^+ \,\forall\, i \neq j \in$

$\{1, 2, \ldots, M\}$, and $\sum_{i=1}^{M} n_i = N + 1$.

$$
\mathbf{Q} = \begin{pmatrix}
\overbrace{\begin{matrix} -\gamma_1 & & \\ \gamma_1 & \ddots & \\ & \ddots & -\gamma_1 \\ & & \gamma_1 \end{matrix}}^{n_1} & & & \\
& \overbrace{\begin{matrix} -\gamma_2 & & \\ \gamma_2 & \ddots & \\ & \ddots & -\gamma_2 \\ & & \gamma_2 \end{matrix}}^{n_2} & \mathbf{0} & \\
& & \overbrace{\begin{matrix} \ddots & \\ \ddots & \ddots \end{matrix}}^{\sum_{i=3}^{M-1} n_i} & \\
& \mathbf{0} & & \overbrace{\begin{matrix} -\gamma_M & & & \\ \gamma_M & \ddots & & \\ & \ddots & -\gamma_M & \\ & & \gamma_M & 0 \end{matrix}}^{n_M}
\end{pmatrix} \tag{27}
$$

1.3.1 Solution

Let us find solutions to (3) using the Laplace transform method. Again defining $\mathbf{B}(s) = s\mathbb{1} - \mathbf{Q}$, where $s \neq -\gamma_i \, \forall \, i \in \{1, \ldots, M\}$, and following the same procedure in §1.2.2, we can write down

$$
[(s\mathbb{1} - \mathbf{Q})^{-1}\boldsymbol{\pi}(0)]_I = \begin{cases}
\left(\dfrac{\gamma_1}{s+\gamma_1}\right)^{k_I} \gamma_1^{-1} & I \neq N+1,\, m_I = 1 \ , \\
\left(\dfrac{\gamma_{m_I}}{s+\gamma_{m_I}}\right)^{k_I} \gamma_{m_I}^{-1} \displaystyle\prod_{j=1}^{m_I - 1} \left(\dfrac{\gamma_j}{s+\gamma_j}\right)^{n_j} & I \neq N+1,\, m_I \neq 1 \ , \\
\dfrac{1}{s} \displaystyle\prod_{j=1}^{M} \left(\dfrac{\gamma_j}{s+\gamma_j}\right)^{n_j} & I = N+1 \ ,
\end{cases}
$$

where $m_I \in \{1, 2, \ldots, M\}$ and $k_I \in \{1, 2, \ldots, n_{m_I}\}$ are counting variables defined by the relationship

$$
\sum_{j=0}^{m_I - 1} n_j + k_I = I \ , \quad n_0 := 0 \ . \tag{28}
$$

Then to calculate $\pi_I(t)$, we notice that $\tilde{f}(s) = (s+\gamma_{m_I})^{-k_I}\prod_{j=1}^{m_I-1}(s+\gamma_j)^{-n_j}$ has poles at $s = -\gamma_1, \ldots, -\gamma_{m_I-1}, -\gamma_{m_I}$, of order $n = n_1, \ldots, n_{m_I-1}, k_I$ respectively, and so

$$\begin{aligned}
\hat{\mathscr{L}}^{-1}\{F(s)\}(t) &= \sum_{j=1}^{m_I} \operatorname{Res}[\tilde{f}(s)\mathrm{e}^{st}, -\gamma_j] \\
&= \sum_{j=1}^{m_I-1} \frac{1}{(n_j-1)!} \lim_{s\to-\gamma_j} \frac{\mathrm{d}^{n_j-1}}{\mathrm{d}s^{n_j-1}} \left(\mathrm{e}^{st} \frac{(s+\gamma_j)^{n_j}}{(s+\gamma_{m_I})^{k_I}} \prod_{k=1}^{m_I-1} (s+\gamma_k)^{-n_k} \right) \\
&\quad + \frac{1}{(k_I-1)!} \lim_{s\to-\gamma_{m_I}} \frac{\mathrm{d}^{k_I-1}}{\mathrm{d}s^{k_I-1}} \left(\mathrm{e}^{st} \frac{(s+\gamma_{m_I})^{k_I}}{(s+\gamma_{m_I})^{k_I}} \prod_{k=1}^{m_I-1} (s+\gamma_k)^{-n_k} \right) \\
&= \sum_{j=1}^{m_I-1} \frac{1}{(n_j-1)!} \lim_{s\to-\gamma_j} \frac{\mathrm{d}^{n_j-1}}{\mathrm{d}s^{n_j-1}} \left(\frac{\mathrm{e}^{st}}{(s+\gamma_{m_I})^{k_I}} \prod_{\substack{k=1\\k\neq j}}^{m_I-1} (s+\gamma_k)^{-n_k} \right) \\
&\quad + \frac{1}{(k_I-1)!} \lim_{s\to-\gamma_{m_I}} \frac{\mathrm{d}^{k_I-1}}{\mathrm{d}s^{k_I-1}} \left(\mathrm{e}^{st} \prod_{k=1}^{m_I-1} (s+\gamma_k)^{-n_k} \right) .
\end{aligned} \tag{29}$$

Now consider function $g(s)$ such that

$$\begin{aligned}
\mathrm{e}^{st}(s+\gamma_{m_I})^{-k_I} \prod_{\substack{k=1\\k\neq j}}^{m_I-1} (s+\gamma_k)^{-n_k} &= \exp\left(st - \sum_{\substack{k=1\\k\neq j}}^{m_I-1} n_k \ln(s+\gamma_k) - k_I \ln(s+\gamma_{m_I}) \right) \\
&=: \mathrm{e}^{g(s)} ,
\end{aligned} \tag{30}$$

and using a simplified form of Faá di Bruno's formula

$$\frac{\mathrm{d}^p}{\mathrm{d}s^p}\mathrm{e}^{g(s)} = \mathrm{e}^{g(s)} B_p\left(g'(s), g''(s), \ldots, g^{(p)}(s)\right) , \tag{31}$$

where $B_p(x_1, x_2, \ldots, x_p)$ is the pth-complete Bell polynomial. If we define a $p\times p$ matrix $\mathbf{M}_p(x_1, \ldots, x_p)$ such that

$$[\mathbf{M}_p]_{ij} = \begin{cases} \binom{p-i}{j-i} x_{j-i+1} & i \leq j , \\ -1 & i = j+1 , \\ 0 & \text{otherwise,} \end{cases} \tag{32}$$

then we can use the identity

$$B_p(x_1, x_2, \ldots, x_p) = \det \mathbf{M}_p(x_1, x_2, \ldots, x_p) \tag{33}$$

and so finally

$$\begin{aligned}
\hat{\mathscr{L}}^{-1}\left\{ \frac{\mathrm{e}^{st}}{(s+\gamma_{m_I})^{k_I}} \prod_{j=1}^{m_I-1} (s+\gamma_j)^{-n_j} \right\} &= \sum_{j=1}^{m_I-1} \frac{\mathrm{e}^{-\gamma_j t} \det \mathbf{H}_{I,j}(t)}{(\gamma_{m_I}-\gamma_j)^{k_I}(n_j-1)!} \prod_{\substack{k=0\\k\neq j}}^{m_I-1} (\gamma_k-\gamma_j)^{-n_k} \\
&\quad + \frac{\mathrm{e}^{-\gamma_{m_I} t} \det \tilde{\mathbf{H}}_I(t)}{(k_I-1)!} \prod_{k=1}^{m_I-1} (\gamma_k-\gamma_{m_I})^{-n_k} .
\end{aligned} \tag{34}$$

Here $\mathbf{H}_{I,j}(t) = 1$ if $n_j = 1$, while if $n_j > 1$, $\mathbf{H}_{I,j}(t)$ is a $(n_j-1)\times(n_j-1)$ matrix defined by

$$[\mathbf{H}_{I,j}]_{pq}(t) = \begin{cases} \dfrac{(n_j-1-p)!}{(n_j-1-q)!} \left(\displaystyle\sum_{\substack{k=0\\k\neq j}}^{m_I-1} \frac{n_k}{(\gamma_j-\gamma_k)^{q-p+1}} + \frac{k_I}{(\gamma_j-\gamma_{m_I})^{q-p+1}} \right) + t\delta_{pq} & p \leq q , \\ -1 & p = q+1 , \\ 0 & \text{otherwise.} \end{cases}$$

Also, $\tilde{\mathbf{H}}(t) = 1$ if $k_I = 1$, while if $k_I > 1$, $\tilde{\mathbf{H}}(t)$ is a $(k_I - 1) \times (k_I - 1)$ matrix defined by

$$[\tilde{\mathbf{H}}_I]_{pq}(t) = \begin{cases} \dfrac{(k_I-1-p)!}{(k_I-1-q)!} \displaystyle\sum_{k=1}^{m_I-1} \frac{n_k}{(\gamma_{m_I}-\gamma_k)^{q-p+1}} + t\delta_{pq} & p \le q \ , \\ -1 & p = q+1 \ , \\ 0 & \text{otherwise.} \end{cases} \tag{35}$$

This gives the overall solution

$$\pi_I(t) = \begin{cases} \mathrm{e}^{-\gamma_1 t} \dfrac{(\gamma_1 t)^{k_I-1}}{(k_I-1)!} & I \ne N+1,\, m_I = 1 \ , \\ \displaystyle\sum_{j=1}^{m_I-1} \frac{\gamma_{m_I}^{k_I-1}\gamma_j^{n_j}\mathrm{e}^{-\gamma_j t}\det \mathbf{H}_{I,j}(t)}{(\gamma_{m_I}-\gamma_j)^{k_I}(n_j-1)!} \prod_{\substack{k=0\\k\ne j}}^{m_I-1} \left(\frac{\gamma_k}{\gamma_k-\gamma_j}\right)^{n_k} & \\ \quad + \dfrac{\gamma_{m_I}^{k_I-1}\mathrm{e}^{-\gamma_{m_I} t}\det \tilde{\mathbf{H}}_I(t)}{(k_I-1)!} \displaystyle\prod_{k=1}^{m_I-1} \left(\frac{\gamma_k}{\gamma_k-\gamma_{m_I}}\right)^{n_k} & I \ne N+1,\, m_I \ne 1 \ , \\ \displaystyle\prod_{k=1}^{M} \gamma_k^{n_k} \times \sum_{j=1}^{M+1} \frac{\mathrm{e}^{-\gamma_j t}\det \mathbf{H}_{N+1,j}(t)}{(n_j-1)!} \prod_{\substack{k=1\\k\ne j}}^{M+1} \left(\frac{1}{\gamma_k-\gamma_j}\right)^{n_k} & I = N+1 \ , \end{cases} \tag{36}$$

where we define $\gamma_0 := 1$, $\gamma_{M+1} := 0$, $n_{M+1} := 1$, $m_{N+1} := M+2$ and $k_{N+1} := 0$ for simplicity of notation.

1.3.2 Derivatives

An analytic expression also allows us to calculate derivatives in a similar way to §1.2.3. Taking a derivative of the Laplace transform, we get

$$\partial_r \tilde{\pi}_I = \begin{cases} \dfrac{n_r}{\gamma_r}\tilde{\pi}_I - \dfrac{n_r}{s+\gamma_r}\tilde{\pi}_I & r < m_I \ , \\ \dfrac{k_I-1}{\gamma_{m_I}}\tilde{\pi}_I - \dfrac{k_I}{s+\gamma_{m_I}}\tilde{\pi}_I & r = m_I \ , \\ 0 & \text{otherwise.} \end{cases}$$

So we need to calculate expressions of the form $\hat{\mathscr{L}}^{-1}\{(s+\gamma_r)^{-1}\tilde{\pi}_j(s)\}$. Rather than use the convolution theorem as before, here we apply the residue theorem multiple times. The first application gives

$$\begin{aligned} \hat{\mathscr{L}}^{-1}\left\{\frac{\tilde{\pi}_I}{s+\gamma_r}\right\} &= \frac{\gamma_{m_I}^{k_I-1}\mathrm{e}^{-\gamma_r t}\det \tilde{\mathbf{K}}_{I,r}(t)}{(\gamma_{m_I}-\gamma_r)^{k_I} n_r!} \prod_{\substack{k=1\\k\ne r}}^{m_I-1} \left(\frac{\gamma_k}{\gamma_k-\gamma_r}\right)^{n_k} \\ &\quad + \sum_{\substack{j=1\\j\ne r}}^{m_I-1} \frac{\gamma_{m_I}^{k_I-1}\gamma_j^{n_j}\mathrm{e}^{-\gamma_j t}\det(\mathbf{H}_{I,j}(t)+\mathbf{K}_{I,r}(n_j))}{(\gamma_{m_I}-\gamma_j)^{k_I}(\gamma_r-\gamma_j)(n_j-1)!} \prod_{\substack{k=0\\k\ne j}}^{m_I-1} \left(\frac{\gamma_k}{\gamma_k-\gamma_j}\right)^{n_k} \\ &\quad + \frac{\gamma_{m_I}^{k_I-1}\mathrm{e}^{-\gamma_{m_I} t}\det(\tilde{\mathbf{H}}_I(t)+\mathbf{K}_{I,r}(k_I))}{(\gamma_r-\gamma_{m_I})(k_I-1)!} \prod_{k=1}^{m_I-1} \left(\frac{\gamma_k}{\gamma_k-\gamma_{m_I}}\right)^{n_k} \ , \end{aligned} \tag{37}$$

where $\tilde{\mathbf{K}}_{I,r}(t)$ is a $n_r \times n_r$ matrix with components

$$[\tilde{\mathbf{K}}_{I,r}]_{pq}(t) = \begin{cases} \dfrac{(n_r-p)!}{(n_r-q)!} \left(\displaystyle\sum_{\substack{k=0\\k\ne r}}^{m_I-1} \frac{n_k}{(\gamma_r-\gamma_k)^{q-p+1}} + \frac{k_I}{(\gamma_r-\gamma_{m_I})^{q-p+1}} \right) + t\delta_{pq} & p \le q \ , \\ -1 & p = q+1 \ , \\ 0 & \text{otherwisem} \end{cases}$$

and where $\mathbf{K}_{I,r}(a) = 0$ if $a = 1$ or if $a > 1$ $\mathbf{K}_{I,r}(a)$ is a $(a-1) \times (a-1)$ matrix with components

$$[\mathbf{K}_{I,r}]_{pq}(n_j) = \begin{cases} \dfrac{(n_j-1-p)!}{(n_j-1-q)!}\dfrac{1}{(\gamma_j-\gamma_r)^{q-p+1}} & p \le q \ , \\ 0 & \text{otherwise,} \end{cases}$$

and

$$[\mathbf{K}_{I,r}]_{pq}(k_I) = \begin{cases} \dfrac{(k_I-1-p)!}{(k_I-1-q)!}\dfrac{1}{(\gamma_{m_I}-\gamma_r)^{q-p+1}} & p \le q \ , \\ 0 & \text{otherwise.} \end{cases}$$

The second application of the residue theorem gives

$$\begin{aligned} \hat{\mathscr{L}}^{-1}\left\{\frac{\tilde{\pi}_I}{s+\gamma_{m_I}}\right\} &= \sum_{j=1}^{m_I-1} \frac{\gamma_{m_I}^{k_I-1}\gamma_j^{n_j}\mathrm{e}^{-\gamma_j t}\det(\mathbf{H}_{I,j}(t)+\mathbf{K}_{I,m_I}(n_j))}{(\gamma_{m_I}-\gamma_j)^{k_I+1}(n_j-1)!}\prod_{\substack{k=0\\k\neq j}}^{m_I-1}\left(\frac{\gamma_k}{\gamma_k-\gamma_j}\right)^{n_k} \\ &\quad + \frac{\gamma_{m_I}^{k_I-1}\mathrm{e}^{-\gamma_{m_I}t}\det\tilde{\mathbf{H}}_{I1}(t)}{k_I!}\prod_{k=1}^{m_I-1}\left(\frac{\gamma_k}{\gamma_k-\gamma_{m_I}}\right)^{n_k} \ , \end{aligned} \tag{38}$$

where $\tilde{\mathbf{H}}_{I1}(t)$ is a $k_I \times k_I$ matrix defined by

$$[\tilde{\mathbf{H}}_{I1}]_{pq}(t) = \begin{cases} \dfrac{(k_I-p)!}{(k_I-q)!}\displaystyle\sum_{k=1}^{m_I-1}\frac{n_k}{(\gamma_{m_I}-\gamma_k)^{q-p+1}} + t\delta_{pq} & p \le q \ , \\ -1 & p = q+1 \ , \\ 0 & \text{otherwise.} \end{cases} \tag{39}$$

This gives the full final expression as

$$
\partial_r \pi_I(t) = \begin{cases}
-t\pi_I(t) + t\pi_{I-1}(t) & r = m_I = 1,\, I \neq N+1\ , \\
\dfrac{k_I-1}{\gamma_{m_I}}\pi_I(t) - k_I\gamma_{m_I}^{k_I-1}\displaystyle\prod_{k=1}^{m_I-1}\gamma_k^{n_k}\times & \\
\quad \left\{ \displaystyle\sum_{j=1}^{m_I-1} \dfrac{\mathrm{e}^{-\gamma_j t}\det(\mathbf{H}_{I,j}(t)+\mathbf{K}_{I,m_I}(n_j))}{(\gamma_{m_I}-\gamma_j)^{k_I+1}(n_j-1)!}\prod_{\substack{k=0\\k\neq j}}^{m_I-1}\left(\dfrac{1}{\gamma_k-\gamma_j}\right)^{n_k}\right. & \\
\quad \left. + \dfrac{\mathrm{e}^{-\gamma_{m_I}t}\det\tilde{\mathbf{H}}_{I1}(t)}{k_I!}\displaystyle\prod_{k=1}^{m_I-1}\left(\dfrac{1}{\gamma_k-\gamma_{m_I}}\right)^{n_k}\right\} & r = m_I \neq 1,\, I\neq N+1\ , \\
\dfrac{n_r}{\gamma_r}\pi_I(t) - n_r k_I\gamma_{m_I}^{k_I-1}\displaystyle\prod_{k=1}^{m_I-1}\gamma_k^{n_k}\times & \\
\quad \left\{ \dfrac{\mathrm{e}^{-\gamma_r t}\det\tilde{\mathbf{K}}_{I,r}(t)}{(\gamma_{m_I}-\gamma_r)^{k_I}n_r!}\displaystyle\prod_{\substack{k=1\\k\neq r}}^{m_I-1}\left(\dfrac{1}{\gamma_k-\gamma_r}\right)^{n_k}\right. & \\
\quad + \displaystyle\sum_{\substack{j=1\\j\neq r}}^{m_I-1} \dfrac{\mathrm{e}^{-\gamma_j t}\det(\mathbf{H}_{I,j}(t)+\mathbf{K}_{I,r}(n_j))}{(\gamma_{m_I}-\gamma_j)^{k_I}(\gamma_r-\gamma_j)(n_j-1)!}\prod_{\substack{k=0\\k\neq j}}^{m_I-1}\left(\dfrac{1}{\gamma_k-\gamma_j}\right)^{n_k} & \\
\quad \left. + \dfrac{\mathrm{e}^{-\gamma_{m_I}t}\det(\tilde{\mathbf{H}}_I(t)+\mathbf{K}_{I,r}(k_I))}{(\gamma_r-\gamma_{m_I})(k_I-1)!}\displaystyle\prod_{k=1}^{m_I-1}\left(\dfrac{1}{\gamma_k-\gamma_{m_I}}\right)^{n_k}\right\} & r < m_I,\, I\neq N+1\ , \\
\dfrac{n_r}{\gamma_r}\pi_{N+1}(t) - n_r\displaystyle\prod_{k=1}^{M+1}\gamma_k^{n_k}\times & \\
\quad \left\{ \mathrm{e}^{-\gamma_r t}\dfrac{\det\tilde{\mathbf{K}}_{N+1,r}(t)}{n_r!}\displaystyle\prod_{\substack{k=1\\k\neq r}}^{M+1}\left(\dfrac{1}{\gamma_k-\gamma_r}\right)^{n_k}\right. & \\
\quad \left. + \displaystyle\sum_{\substack{j=1\\j\neq r}}^{M+1} \mathrm{e}^{-\gamma_j t}\dfrac{\det(\mathbf{H}_{N+1,j}(t)+\mathbf{K}_{N+1,r}(n_j))}{(\gamma_r-\gamma_j)(n_j-1)!}\prod_{\substack{k=0\\k\neq j}}^{M+1}\left(\dfrac{1}{\gamma_k-\gamma_j}\right)^{n_k}\right\} & I = N+1\ , \\
0 & \text{otherwise.}
\end{cases}
\tag{40}
$$

2 Metric for the shedding models

2.1 Underlying Fisher-Rao metric

We first note the straightforwardly-obtained result that if ϕ is the pdf of a normal distribution with mean $\mu(\boldsymbol{\theta})$ and standard deviation $\sigma(\boldsymbol{\theta})$, then the Fisher-Rao metric will contain terms like

$$
g_{a,b} = \mathbb{E}_\phi[\partial_a \ln(\phi)\partial_b \ln(\phi)] = \frac{1}{\sigma^2}\partial_a\mu\partial_b\mu + \frac{2}{\sigma^2}\partial_a\sigma\partial_b\sigma\ . \tag{41}
$$

We now consider how this metric is calculated for the four different shedding models considered in the main paper.

2.2 The SIR model

For the simple SIR model defined in the main paper (Eq. (14)) we have

$$\mu(\tau,\gamma) = \tau \mathrm{e}^{-\gamma} \ , \qquad \sigma(\tau,\gamma) = \sigma \ . \tag{42}$$

The derivatives are therefore

$$\partial_\tau \mu = \mathrm{e}^{-\gamma} \ , \quad \partial_\gamma \mu = -\tau \mathrm{e}^{-\gamma} \ , \quad \partial_a \sigma = 0 \ . \tag{43}$$

Substituting into (41) gives the Fisher-Rao metric as having the following form:

$$\mathbf{G} = \frac{\mathrm{e}^{-2\gamma}}{\sigma^2} \begin{pmatrix} 1 & -\tau \\ -\tau & \tau^2 \end{pmatrix} \ . \tag{44}$$

This has the issue that there is full unidentifiability, reflected in the fact that this metric attributes zero distance to travel along constant-$\tau\mathrm{e}^{-\gamma}$ curves. Our solution to this problem is to add an amount of distance in these directions to give full metric

$$\mathbf{G}_\alpha = \frac{\mathrm{e}^{-2\gamma}}{\sigma^2} \left\{ \begin{pmatrix} 1 & -\tau \\ -\tau & \tau^2 \end{pmatrix} + \alpha \begin{pmatrix} \tau^2 & -\tau \\ -\tau & 1 \end{pmatrix} \right\} \ , \tag{45}$$

where α is a constant parameterising the amount of distance added.

While it was not necessary to do this for any of our other models, we suggest that as a general methodological point if an initial metric took a form similar to (45), but for very small α that caused potential numerical issues with forming its inverse, then it may be advisable to increase the value of α by hand.

2.3 Influenza and Ebola

Here our likelihood is given by a product of normal probability density functions

$$L = \prod_{t\in\mathcal{T}} \phi(\mu(t), \sigma(t)) \ , \tag{46}$$

where

$$\mu(t) = \tau \sum_{i=1}^{m} \pi_i(t,\gamma) =: \tau\overline{\pi}(t) \ , \quad \sigma(t) = \sigma_t \text{ is given in data.} \tag{47}$$

Given the results above for $\pi_i(t)$, we can then write down that for influenza the metric has components

$$g_{\tau,\tau} = 0 \ , \quad g_{i,j} = \sum_{t\in\mathcal{T}} \frac{\tau}{\sigma_t^2} \partial_i \overline{\pi}(t) \partial_j \overline{\pi}(t) \ , \quad g_{\tau,i} = \sum_{t\in\mathcal{T}} \frac{\overline{\pi}(t)}{\sigma_t^2} \partial_i \overline{\pi}(t) \ . \tag{48}$$

For Ebola, we have to consider both high- and low-viraemic pathways of infection separately, but otherwise the metric is

2.4 Norovirus

Our norovirus model has the same form as (46) except that $\sigma(t) = \sigma$ is a model parameter.

Since for these data we have a large number of approximately uniformly-distributed time points, we use integrals rather than sums over time for computational efficiency leading to expressions

$$\begin{aligned} g_{i,j} &= \int_0^\infty \frac{\tau}{\sigma^2} \partial_i \overline{\pi}(t) \partial_j \overline{\pi}(t) \mathrm{d}t \ , & g_{\tau,i} &= \int_0^\infty \frac{\overline{\pi}(t)}{\sigma^2} \partial_i \overline{\pi}(t) \mathrm{d}t \ , \\ g_{\sigma,\sigma} &= \frac{2}{\sigma^2} \ , & g_{\tau,\tau} &= g_{\sigma,a\neq\sigma} = 0 \ , \end{aligned} \tag{49}$$

which can be straightforwardly computed from the results above.

2.5 Other contributions to the metric

We can include the contribution of a rate-ρ_a exponential prior on θ_a through performing calculations for uniform / improper priors and then making the transformation

$$\partial_a \mathcal{L} \to \partial_a \mathcal{L} - \rho_a \ . \tag{50}$$

For SMMALA the metrics we have considered so far are all that is required, and have the primary benefit of introducing local second-order derivative information into the MCMC algorithm, but for WLMC we make additional use of the possibilities for reduction of global distances possible in a geometric approach.

3 The WLMC algorithm

3.1 Lagrangian Monte Carlo (LMC)

The 'HMC' dynamics introduced in the main paper can be generalised to a geometric approach in two ways. The 'RMHMC' approach of [2] involves *Hamiltonian* dynamics with a discretized integrator, the generalized leapfrog method [6, 2], which requires significant numerical effort, in particular fixed-point iterations, to solve implicit equations. This step is potentially computational intensive (repeated matrix inversion of $\mathbf{G}(\boldsymbol{\theta})$ involves $O(D^{2.373})$ operations in dimension D), and can sometimes be numerically unstable [3].

To address this issue, Lan et al. [4] propose an explicit integrator for geometric MCMC by using the following *Lagrangian* dynamics:

$$\frac{\mathrm{d}\boldsymbol{\theta}}{\mathrm{d}t} = \mathbf{v} \ , \qquad \frac{\mathrm{d}\mathbf{v}}{\mathrm{d}t} = \mathbf{G}(\boldsymbol{\theta})^{-1}\boldsymbol{\partial}\mathcal{L} - \mathbf{v}^{\mathsf{T}}\boldsymbol{\Gamma}(\boldsymbol{\theta})\mathbf{v} \ , \tag{51}$$

where $\mathbf{v}(0) := \mathbf{G}(\boldsymbol{\theta}(0))^{-1}\mathbf{p}(0) \sim \mathcal{N}(\mathbf{0}, \mathbf{G}(\boldsymbol{\theta}(0))^{-1})$, and $\boldsymbol{\Gamma}(\boldsymbol{\theta})$ are Christoffel Symbol of the second kind whose (i,j,k)-th element is $\Gamma^k_{ij} = \frac{1}{2}g^{km}(\partial_i g_{mj} + \partial_j g_{im} - \partial_m g_{ij})$ with g^{km} being the (k,m)-th element of $\mathbf{G}(\boldsymbol{\theta})^{-1}$.

The following explicit integrator can then be derived for these dynamics:

$$\mathbf{v}^{(\ell+\frac{1}{2})} = \left[\mathbf{I} + \frac{\varepsilon}{2}\boldsymbol{\Omega}(\boldsymbol{\theta}^{(\ell)}, \mathbf{v}^{(\ell)}))\right]^{-1}\left[\mathbf{v}^{(\ell)} - \frac{\varepsilon}{2}\mathbf{G}(\boldsymbol{\theta}^{(\ell)})^{-1}\nabla_{\boldsymbol{\theta}}\phi(\boldsymbol{\theta}^{(\ell)})\right] \ , \tag{52}$$

$$\boldsymbol{\theta}^{(\ell+1)} = \boldsymbol{\theta}^{(\ell)} + \varepsilon\mathbf{v}^{(\ell+\frac{1}{2})} \ , \tag{53}$$

$$\mathbf{v}^{(\ell+1)} = \left[\mathbf{I} + \frac{\varepsilon}{2}\boldsymbol{\Omega}(\boldsymbol{\theta}^{(\ell+1)}, \mathbf{v}^{(\ell+\frac{1}{2})}))\right]^{-1}\left[\mathbf{v}^{(\ell+\frac{1}{2})} - \frac{\varepsilon}{2}\mathbf{G}(\boldsymbol{\theta}^{(\ell+1)})^{-1}\nabla_{\boldsymbol{\theta}}\phi(\boldsymbol{\theta}^{(\ell+1)})\right] \ , \tag{54}$$

where $\Omega(\boldsymbol{\theta}^{(\ell)}, \mathbf{v}^{(\ell)}))_{kj} := (v^{(\ell)})^i\Gamma(\boldsymbol{\theta}^{(\ell)})^k_{ij}$. Such an integrator is time reversible but not volume preserving. The acceptance probability is adjusted to have the detailed balance condition hold [4]:

$$\alpha = \min\left\{1, \exp(-E(\mathbf{z}^{(L+1)}) + E(\mathbf{z}^{(1)}))\left|\frac{d\mathbf{z}^{(L+1)}}{d\mathbf{z}^{(1)}}\right|\right\} \ , \tag{55}$$

where the Jacobian determinant is

$$\left|\frac{d\mathbf{z}^{(\ell+1)}}{d\mathbf{z}^{(\ell)}}\right| = \frac{\det(\mathbf{I} - \varepsilon/2\boldsymbol{\Omega}(\boldsymbol{\theta}^{(\ell+1)}, \mathbf{v}^{(\ell+1)}))\det(\mathbf{I} - \varepsilon/2\boldsymbol{\Omega}(\boldsymbol{\theta}^{(\ell)}, \mathbf{v}^{(\ell+1/2)}))}{\det(\mathbf{I} + \varepsilon/2\boldsymbol{\Omega}(\boldsymbol{\theta}^{(\ell+1)}, \mathbf{v}^{(\ell+1/2)}))\det(\mathbf{I} + \varepsilon/2\boldsymbol{\Omega}(\boldsymbol{\theta}^{(\ell)}, \mathbf{v}^{(\ell)}))} \ , \tag{56}$$

and $E(\mathbf{z})$ is the *energy* for the Lagrangian dynamics defined as:

$$E(\boldsymbol{\theta}, \mathbf{p}) = -\log\pi(\boldsymbol{\theta}|\mathcal{D}) - \frac{1}{2}\log\det\mathbf{G}(\boldsymbol{\theta}) + \frac{1}{2}\mathbf{v}^{\mathsf{T}}\mathbf{G}(\boldsymbol{\theta})\mathbf{v} \ . \tag{57}$$

The resulting algorithm, *Lagrangian Monte Carlo* (LMC) is a valid exact sampler and has the same strength in exploring complex geometry as RHMC does. LMC is sometimes more efficient and stable than RHMC – for more details see [3, 4].

3.2 WLMC

When the target distribution is multi-modal, derivative-based and geometric algorithms tend to fail as they are easily trapped in some of the modes without visiting all of them. Making proposals by numerically simulating Hamiltonian dynamics, the sampler has difficulty in passing through low probability regions [7]. Compared to HMC, the geometric RHMC and LMC methods perform even worse in relation to this issue because they are more adapted to the local geometry and more likely to be trapped in one mode.

To overcome this issue, some kind of global knowledge of the distribution needs to learned and incorporated. Lan et al. [5] proposed the idea of using *wormholes* for these geometric algorithms (HMC/RHMC/LMC) to work on multi-modal distributions. The proposed method comes in 2 parts: a distance-shortening metric and a mode-jumping mechanism.

3.3 Wormhole Metric

Let $\hat{\boldsymbol{\theta}}_1$ and $\hat{\boldsymbol{\theta}}_2$ be two modes of the target distribution. We define a straight line segment, $\mathbf{v}_W := \hat{\boldsymbol{\theta}}_2 - \hat{\boldsymbol{\theta}}_1$, and refer to a small neighborhood (tube) of the line segment as a *wormhole*. Next, we define a *wormhole metric*, $\mathbf{G}_W(\boldsymbol{\theta})$, in the vicinity of the wormhole. For a pair of tangent vectors $\mathbf{u}, \mathbf{w}$ at $\boldsymbol{\theta}$, *wormhole metric* $\mathbf{G}_W$ is defined as follows

$$\mathbf{G}_W^*(\mathbf{u},\mathbf{w}) := \langle \mathbf{u} - \langle \mathbf{u}, \mathbf{v}_W^* \rangle \mathbf{v}_W^*, \mathbf{w} - \langle \mathbf{w}, \mathbf{v}_W^* \rangle \mathbf{v}_W^* \rangle = \mathbf{u}^{\mathsf{T}}[\mathbf{I} - \mathbf{v}_W^* (\mathbf{v}_W^*)^{\mathsf{T}}]\mathbf{w} \ , \tag{58}$$

$$\mathbf{G}_W = \mathbf{G}_W^* + \varepsilon \mathbf{v}_W^* (\mathbf{v}_W^*)^{\mathsf{T}} = \mathbf{I} - (1-\varepsilon)\mathbf{v}_W^* (\mathbf{v}_W^*)^{\mathsf{T}} \ , \tag{59}$$

where $\mathbf{v}_W^* = \mathbf{v}_W / \|\mathbf{v}_W\|$, and $0 < \varepsilon \ll 1$ is a small positive number. To see that $\mathbf{G}_W$ in fact shortens the distance between $\hat{\boldsymbol{\theta}}_1$ and $\hat{\boldsymbol{\theta}}_2$, consider a simple case of a straight line: $\boldsymbol{\theta}(t) = \hat{\boldsymbol{\theta}}_1 + \mathbf{v}_W t, t \in [0,1]$. In this case, the distance under $\mathbf{G}_W$ is

$$\mathrm{dist}(\hat{\boldsymbol{\theta}}_1, \hat{\boldsymbol{\theta}}_2) = \int_0^1 \sqrt{\mathbf{v}_W^{\mathsf{T}} \mathbf{G}_W \mathbf{v}_W} dt = \sqrt{\varepsilon}\|\mathbf{v}_W\| \ll \|\mathbf{v}_W\| \ ,$$

which is much smaller than the Euclidean distance.

Next, we define the overall metric, $\mathbf{G}$, for the whole parameter space of $\boldsymbol{\theta}$ as a weighted sum of the base metric $\mathbf{G}_0$ and the wormhole metric $\mathbf{G}_W$,

$$\mathbf{G}(\boldsymbol{\theta}) = (1 - \mathfrak{m}(\boldsymbol{\theta}))\mathbf{G}_0(\boldsymbol{\theta}) + \mathfrak{m}(\boldsymbol{\theta})\mathbf{G}_W \ , \tag{60}$$

where $\mathfrak{m}(\boldsymbol{\theta}) \in (0,1)$ is a mollifying function designed to make the wormhole metric $\mathbf{G}_W$ influential in the vicinity of the wormhole only.

3.4 Wormhole Network

For more than two modes, the above method alone could suffer from two potential shortcomings in higher dimensions. First, the effect of wormhole metric could diminish quickly as the sampler leaves one mode and moves towards another mode. Secondly, such a mechanism, which modifies the dynamics in the existing parameter space, could interfere with the native dynamics in the neighborhood of a

wormhole, possibly preventing the sampler from properly exploring areas around the modes as well as some low probability regions.

To address the first issue, we add an external vector field to enforce the movement between modes. More specifically, we define a vector field, $\mathbf{f}(\boldsymbol{\theta}, \mathbf{v})$, in terms of the position parameter $\boldsymbol{\theta}$ and the velocity vector $\mathbf{v} = \mathbf{G}(\boldsymbol{\theta})^{-1}\mathbf{p}$ as follows:

$$\mathbf{f}(\boldsymbol{\theta}, \mathbf{v}) := \exp\{-V(\boldsymbol{\theta})/(DF)\}U(\boldsymbol{\theta})\langle \mathbf{v}, \mathbf{v}_W^* \rangle \mathbf{v}_W^* = \mathfrak{m}(\boldsymbol{\theta})\langle \mathbf{v}, \mathbf{v}_W^* \rangle \mathbf{v}_W^* \ , \tag{61}$$

with mollifier $\mathfrak{m}(\boldsymbol{\theta}) := \exp\{-V(\boldsymbol{\theta})/(DF)\}$, where D is the dimension, $F > 0$ is the influence factor, and $V(\boldsymbol{\theta})$ is a vicinity function indicating the Euclidean distance from the line segment $\mathbf{v}_W$,

$$V(\boldsymbol{\theta}) := \langle \boldsymbol{\theta} - \hat{\boldsymbol{\theta}}_1, \boldsymbol{\theta} - \hat{\boldsymbol{\theta}}_2 \rangle + |\langle \boldsymbol{\theta} - \hat{\boldsymbol{\theta}}_1, \mathbf{v}_W^* \rangle||\langle \boldsymbol{\theta} - \hat{\boldsymbol{\theta}}_2, \mathbf{v}_W^* \rangle| \ . \tag{62}$$

After adding the vector field, we modify the Hamiltonian/Lagrangian dynamics governing the evolution of $\boldsymbol{\theta}$ as follows:

$$\dot{\boldsymbol{\theta}} = \mathbf{v} + \mathbf{f}(\boldsymbol{\theta}, \mathbf{v}) \ . \tag{63}$$

To address the second issue, we allow the wormholes to pass through an extra auxiliary dimension to avoid their interference with the existing dynamics in the given parameter space. In particular we introduce an auxiliary variable $\theta_{D+1} \sim \mathcal{N}(0, 1)$ corresponding to an auxiliary dimension. We use $\tilde{\boldsymbol{\theta}} := (\boldsymbol{\theta}, \theta_{D+1})$ to denote the position parameters in the resulting $D + 1$ dimensional space $\mathcal{M}^D \times \mathbb{R}$. θ_{D+1} can be viewed as random noise independent of $\boldsymbol{\theta}$ and contributes $\frac{1}{2}\theta_{D+1}^2$ to the total potential energy. Correspondingly, we augment velocity $\mathbf{v}$ with one extra dimension, denoted as $\tilde{\mathbf{v}} := (\mathbf{v}, v_{D+1})$. At the end of the sampling, we project $\tilde{\boldsymbol{\theta}}$ to the original parameter space and discard θ_{D+1}.

We refer to $\mathcal{M}^D \times \{-h\}$ as the *real world*, and call $\mathcal{M}^D \times \{+h\}$ the *mirror world*. Here, h is half of the distance between the two worlds, and it should be in the same scale as the average distance between the modes. For most of the examples discussed here, we set $h = 1$. Figure S1 illustrates how the two worlds are connected by networks of wormholes.

One can refer to [5] for full algorithmic details of Wormhole HMC/LMC, including the case where the modes are initially unknown.

4 Supplementary figure

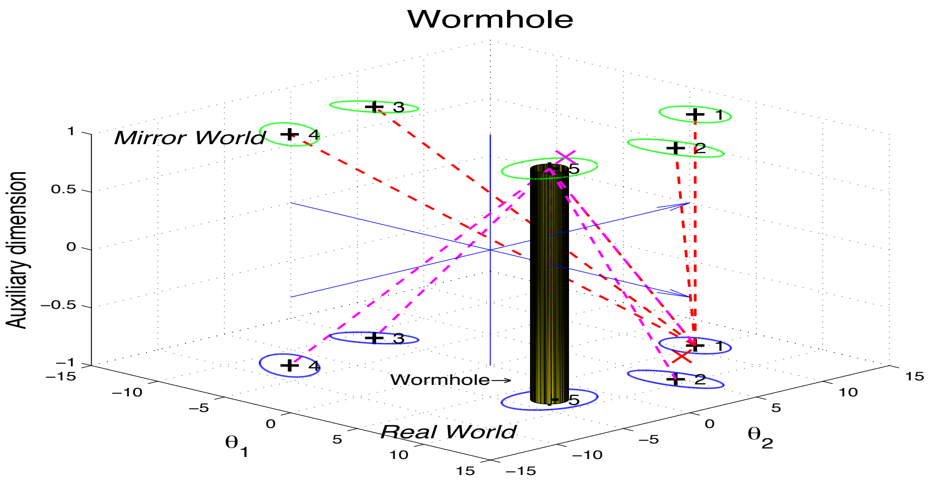

Figure S1: Illustrating a wormhole network connecting the real world to the mirror world ($h = 1$). As an example, the cylinder shows a wormhole connecting mode 5 in the real world to its mirror image. The dashed lines show two sets of wormholes. The red lines shows the wormholes when the sampler is close to mode 1 in the real world, and the magenta lines show the wormholes when the sampler is close to mode 5 in the mirror world.

5 Mathematica code

We provide the following code sample provides as an example of how closed-form expressions for quantities of interest for the influenza model can be obtained using computer algebra.

```
(* Set up the SEEIIR equations *)
seeiirEqs = {
e1'[t] ==-2p[3] e1[t],
e2'[t] ==2p[3](e1[t]- e2[t]),
i1'[t] ==2p[3] e2[t]- 2p[2] i1[t],
i2'[t] ==2p[2](i1[t]- i2[t]),
Rt'[t]==i1[t]+i2[t],
e1[0]==1,
e2[0]==0,
i1[0]==0,
i2[0]==0,
Rt[0]==0};

(* Find a solution, the force of infection and its derivatives *)
sol = DSolve[seeiirEqs,{e1[t],e2[t],i1[t],i2[t],Rt[t]},t];
la = FullSimplify[p[1] (i1[t]+i2[t])/.sol[[1]]]
dla = Table[FullSimplify[D[la,p[k]]],{k,1,3}]
ddla = Table[FullSimplify[D[dla[[l]],p[k]]],{k,1,3},{l,1,3}]
```

References

[1] A. Beskos, F. J. Pinski, J. M. Sanz-Serna, and A. M. Stuart. Hybrid Monte Carlo on Hilbert spaces. *Stochastic Processes and their Applications*, 121(10):2201–2230, 2011.

[2] M. Girolami and B. Calderhead. Riemann manifold Langevin and Hamiltonian Monte Carlo methods. *Journal of the Royal Statistical Society, Series B*, 73(2):123–214, 2011.

[3] S. Lan. Advanced Bayesian computational methods through geometric techniques. PhD thesis, Long Beach, CA, USA. AAI3605182., 2013.

[4] S. Lan, V. Stathopoulos, B. Shahbaba, and M. Girolami. Markov chain Monte Carlo from Lagrangian dynamics. Journal of Computational and Graphical Statistics DOI: 10.1080/10618600.2014.902764, 2014.

[5] S. Lan, J. Streets, and B. Shahbaba. Wormhole Hamiltonian Monte Carlo. AAAI Conference on Artificial Intelligence, 2014.

[6] B. Leimkuhler and S. Reich. *Simulating Hamiltonian Dynamics.* Cambridge University Press, 2005.

[7] C. Sminchisescu and M. Welling. Generalized darting Monte Carlo. *Pattern Recognition*, 44(1011):2738–2748, 2011. Semi-Supervised Learning for Visual Content Analysis and Understanding.
